# Supplementary material for: Assessing the environmental factors affecting the sustainability of Aini Falaj system
Source: PLoS One. 2024 May 14;19(5):e0301832. doi: 10.1371/journal.pone.0301832 (PMC11093386; doi:10.1371/journal.pone.0301832)
Supplement: S1 File — Both influence and Cook’s D values assess the impact of a feature on the estimate of regression coefficients, considering the vitality of Aini Falajs and environmental factors. (PDF) [file pone.0301832.s001.pdf]

Influence	COOKS-D	CND-Number
0.892805266	0.031740447	1441.482671
0.603242009	0.008484154	234.5671201
0.271981936	0.013937635	250.2550718
0.341734715	0.00221756	2148.032605
0.27144402	0.000236538	2208.038854
0.329527412	0.000273619	2213.063609
0.181468637	8.37537E-05	2130.230091
0.451813852	7.05849E-06	2299.06396
0.225369066	0.000322634	2274.47211
0.422127045	0.002141443	2498.427888
0.709384236	0.002667287	1902.039752
0.629351044	0.000154672	351.2054931
0.327357571	5.63259E-08	272.3764242
0.52047336	0.021278607	1776.01505
0.732621845	0.004840476	441.5819414
0.319607528	0.000754266	444.7116282
0.23689665	3.7079E-06	442.6451374
0.157201281	2.40785E-07	442.2192096
0.176586699	6.56092E-06	442.0344917
0.174156991	2.6374E-05	441.9155015
0.246319687	9.46568E-06	442.3994217
0.303941376	0.000352233	439.6914219
0.224781153	3.21026E-05	438.0053364
0.255697933	2.36991E-05	437.3292999
0.260974023	9.53833E-08	436.8837431
0.703331971	0.005409387	2.99131E+16
0.153954849	0.00027166	1.18821E+17
0.341264117	0.00288369	1.92543E+16
0.156530907	0.000336212	4.36112E+16
0.169759442	3.70741E-05	5.47112E+16
0.3776239	0.000169754	9.77443E+16
0.34293471	0.007250987	1.34132E+16
0.140258257	0.000196748	7.45069E+16
0.351364581	0.00050727	1.33862E+16
0.125803126	6.14941E-08	4.92695E+16
0.117249066	3.17649E-07	3.35269E+16
0.149684169	0.00017501	5.63964E+16
0.108007592	8.7436E-05	1.42007E+16
0.181887587	2.27866E-05	2.31637E+17
0.111623873	3.79513E-06	1.10966E+17
0.247238859	1.46583E-06	3.36066E+16
0.170315626	7.36467E-05	3.73635E+16
0.146002165	2.60204E-05	1.16759E+17
0.125097511	2.68509E-06	1.25696E+17
0.205716747	2.10525E-05	2.87448E+16
0.251994762	1.34927E-05	4.39218E+16

0.260456995	0.000146788	3.85941E+16
0.154039534	1.49577E-05	5.3244E+17
0.423601308	0.003723448	5.28009E+16
0.267629276	8.16657E-06	4.22857E+16
0.134012065	5.24183E-05	6.97525E+16
0.127151972	1.1621E-05	4.52456E+16
0.139795666	2.65276E-05	8.11878E+16
0.633325116	0.000555847	4.29311E+16
0.163313423	0.000126877	2.10975E+16
0.15747565	0.000479591	3.38543E+16
0.31935262	0.000313514	1.01727E+17
0.186297537	3.64694E-06	2.1995E+16
0.128119116	4.37153E-06	2.61096E+16
0.467825957	4.06431E-05	4.3102E+16
0.196741906	0.000124708	1.30836E+16
0.4547599	0.000347966	3.92092E+16
0.241706373	0.000246028	1.60893E+16
0.701730153	0.01740241	2.3695E+16
0.444090156	0.001985382	1.11286E+16
0.210498303	0.00301814	6.63329E+16
0.510281663	0.018127167	4.51661E+16
0.403544457	9.42374E-06	2.9097E+16
0.622661552	0.000899896	2.93019E+16
0.20562425	0.000841945	2.13096E+16
0.162503223	1.45601E-05	4.28255E+16
0.213900027	1.01121E-05	4.52884E+16
0.675995331	1.18077E-06	1.40448E+16
0.459421974	0.000755934	3.98194E+16
0.298380567	0.000604114	3.63386E+16
0.46202796	0.000405082	5.67319E+16
0.28382202	5.93515E-05	3.30423E+16
0.216268729	1.66499E-05	1.33612E+17
0.145064315	2.66364E-05	3.49183E+16
0.314925211	0.000147049	3.60637E+16
0.217406874	7.82804E-05	2.4664E+16
0.317714561	0.000225464	5.71963E+16
0.293832782	7.42372E-06	3.4577E+16
0.35656864	0.000301766	492.8255293
0.508181651	0.000624356	741.7837829
0.678560824	0.024620689	852.8103112
0.321283083	4.79718E-07	488.0696705
0.277900916	1.09797E-06	488.9523287
0.349589068	0.005465875	484.269791
0.384590054	0.00359082	480.6765905
0.413074974	0.000352555	522.5971168
0.245591139	7.47133E-05	433.713101
0.679276099	0.011458259	522.018072

0.468989801	0.000134107	440.5650859
0.259541672	9.27691E-05	388.628987
0.221001745	1.33732E-05	333.2009156
0.241779381	0.000325692	365.5716005
0.248257887	0.000174925	2.78165E+16
0.285610668	0.000200505	3.28857E+16
0.301249417	9.70856E-05	1.4459E+16
0.203121967	1.02409E-06	2.98691E+16
0.253628092	7.78549E-06	3.79452E+16
0.193732556	5.85377E-07	1.47317E+16
0.228757661	0.000290708	9.72125E+15
0.712803842	0.000229199	532.6345258
0.528106464	0.000123022	535.6154548
0.655286641	0.000739139	1186.987903
0.206616028	0.000106947	575.6963493
0.25017194	0.000446385	403.1303042
0.244513689	0.000307313	403.2847005
0.246389206	0.000379461	404.4048077
0.409176785	0.002665677	373.9874187
0.389205772	0.002376095	371.7188564
0.304682394	0.000122615	576.3876329
0.341309793	0.000576754	358.2501392
0.368432826	0.000635535	432.7091305
0.396626327	1.52527E-05	581.8216744
0.326998233	5.14115E-08	615.7812659
0.437017491	0.002071525	551.8748375
0.309665987	0.002113389	552.8248081
0.267422161	0.000221238	471.6288841
0.339777671	0.000779923	571.5295701
0.157984921	1.41934E-05	578.375437
0.136640801	1.66448E-05	580.3642017
0.18466073	5.82983E-06	575.1404876
0.174161384	2.23438E-06	581.5885599
0.290615857	0.00016535	578.5478556
0.153709755	3.08417E-05	582.556471
0.284634466	0.000271565	570.5404818
0.402677167	4.26906E-07	584.0847327
0.187742059	2.51944E-05	1.43605E+16
0.340273289	3.35276E-05	3.26585E+16
0.197865884	7.28074E-05	2.88842E+16
0.220709258	5.55024E-05	2.22359E+16
0.233531875	0.000208111	2.54966E+16
0.260441358	0.000448169	4.90498E+16
0.162913272	2.9627E-08	1.64582E+17
0.159362241	4.66665E-05	2.06982E+16
0.200714477	3.12504E-05	2.82509E+16
0.143219167	0.000137682	1.32689E+16

0.641633459	1.18095E-06	2.17643E+16
0.155617456	2.16421E-05	1.50136E+16
0.231017425	1.67247E-05	1.39747E+16
0.534819032	0.00064351	3.63474E+16
0.389651519	6.94542E-05	2.91492E+16
0.249769534	0.000264383	2.40718E+16
0.822049367	0.037599203	1.67845E+16
0.613758813	4.39629E-06	5.0633E+16
0.507751265	0.002830748	5.52504E+16
0.299583569	9.706E-06	1.47676E+16
0.405318141	0.000444758	7.77495E+15
0.410011521	1.32625E-07	1.2158E+16
0.370323478	0.000786181	7.28391E+16
0.69585423	0.010405378	2.57216E+16
0.280268509	0.001026723	9.91588E+16
0.390356322	0.000499852	1.9196E+16
0.326079631	0.000431617	7.81228E+16
0.638374304	7.44154E-05	2.02585E+16
0.360446745	0.00227803	1.18502E+16
0.615017659	0.005773265	1.0694E+17
0.411743006	0.001033954	4.49454E+16
0.329259468	0.000129823	4.62576E+16
0.236685893	5.23139E-06	3.9807E+16
0.23731431	0.00012821	1.34202E+16
0.43115898	0.007070441	3.03298E+16
0.396474	0.000365217	5.94511E+16
0.321448399	7.33753E-05	3.88263E+16
0.455559876	0.005065776	5.45301E+16
0.734507244	0.023918451	165.3549534
0.382529685	0.001034864	149.294076
0.42411609	0.000259516	148.5771468
0.487044983	0.001143364	166.2366915
0.32493178	0.000662725	130.064663
0.429814598	0.001604109	85.97235594
0.461558229	0.011121848	7.7143E+16
0.358415142	0.000231196	85.50745806
0.576290406	0.011222584	202.5439932
0.434435518	0.000622439	1.38352E+16
0.576355421	0.001781982	1.84542E+16
0.455942428	0.000990696	214.9834605
0.420846742	0.002498578	199.1111117
0.480466492	0.000649236	191.4747032
0.358795058	0.000475952	2.56129E+16
0.342555887	0.004861122	1.44158E+17
0.569219015	0.036367597	1.74648E+16
0.226459827	0.0013838	7.9178E+16
0.229713917	0.003183187	1.04853E+17

0.364401805	0.000230934	2.79164E+16
0.388699701	0.002165577	3.52178E+16
0.223649723	0.000978657	101.6058428
0.543284965	0.001503205	114.2575572
0.612884518	0.001639713	175.9124548
0.357955065	0.001420528	133.2435585
0.388248809	0.001345303	2.64865E+16
0.359833932	1.35933E-05	2.14374E+16
0.419369104	0.002563259	2.30532E+16
0.416909789	0.001723292	2.94016E+16
0.569467875	0.000300846	1.98738E+16
0.42923769	0.000126646	2.67405E+16
0.29117573	8.42709E-05	2.08296E+16
0.263028178	0.000340568	2.31258E+16
0.350232675	1.45642E-05	2.07654E+16
0.31882132	0.00196134	1.8903E+16
0.345800421	0.000524651	4.20581E+16
0.330287649	0.002597123	5.39162E+16
0.380146525	0.003125394	2.49333E+16
0.469742539	0.017186664	1.98453E+16
0.480145078	0.011826981	2.96355E+16
0.523472107	0.010244359	5.11712E+16
0.82980294	0.061647161	4.96537E+16
0.625162906	0.009577852	3.92932E+16
0.149935588	0.00026217	1.16452E+16
0.247701186	0.000241405	1.9394E+16
0.259821629	0.000456109	1.19271E+16
0.414846469	0.00045103	1.1334E+16
0.267048474	0.000428728	1.73534E+16
0.182437865	0.000124252	1.02129E+17
0.208066075	8.9831E-05	3.90202E+16
0.186772371	4.80407E-05	3.34407E+16
0.383795561	1.55304E-05	2.32861E+17
0.232814831	0.00036235	1.8836E+16
0.345870082	0.000183089	1.69051E+16
0.331676697	0.000306984	4.58759E+16
0.177192181	1.73644E-05	2.24936E+16
0.206937729	7.05577E-05	2.81832E+16
0.3230325	6.06601E-05	6.58843E+16
0.484137713	0.000130718	2.87538E+16
0.519264254	0.000234497	3.34311E+16
0.227443703	0.000139202	3.98398E+16
0.245618892	0.000493127	1.08648E+16
0.201448767	3.92098E-06	4.21836E+16
0.251015051	0.000200421	7.09821E+16
0.178507748	0.000246834	2.47046E+16
0.676066831	0.003862225	2.48433E+16

0.373954896	0.000197318	1.88275E+16
0.307998502	0.000485529	1.97474E+16
0.279054994	6.49255E-05	3.18001E+16
0.585053934	1.08512E-05	1.0964E+16
0.612781335	0.003042563	3.57311E+16
0.489526576	0.00193125	1.44231E+16
0.595782348	0.000856344	7.09674E+16
0.683800236	0.036698195	142.7892857
0.306720594	0.000237264	6.72241E+16
0.677254213	2.11229E-06	1.80731E+16
0.209526457	0.000163846	3.15789E+16
0.870913474	0.011172258	40.77932225
0.566842527	0.005079044	84.80433667
0.36555776	0.005918521	2.47324E+16
0.416597929	0.0007334	2.39684E+16
0.153281091	0.000164404	2472.936185
0.100019334	0.000145143	2377.793713
0.101142665	2.60916E-05	2395.552276
0.105123046	5.6297E-05	2426.541351
0.09278719	1.43206E-05	2396.651723
0.108247718	5.59009E-05	2377.635808
0.145923873	8.90888E-05	2372.103946
0.14165426	0.000192809	2354.411626
0.157406022	0.000219919	2350.187767
0.084804887	2.84581E-08	2347.821095
0.128442332	5.96949E-07	2344.173969
0.095479468	7.47643E-05	2379.375751
0.102923285	3.86001E-05	2378.300215
0.317919433	0.004983487	2262.172034
0.84586949	0.155707681	1664.524597
0.948740371	0.159679274	953.7209308
0.648402611	0.01033648	770.6066681
0.77885619	0.13742404	8.09628E+16
0.635581613	4.26569E-05	606.1194685
0.580362179	0.001107924	549.3855322
0.678310461	0.005133986	660.0064621
0.611496487	0.225732532	1.07915E+16
0.887098362	0.022361532	627.9830164
0.392381388	0.008698368	2.49208E+16
0.461731467	0.004913893	3.91037E+16
0.416325124	0.01101979	2.25248E+16
0.221316968	0.00082318	2.69828E+16
0.443731236	0.000419728	2.33044E+16
0.345545164	0.000164342	7.04862E+16
0.248478737	0.004541326	3.20953E+16
0.22965508	0.000481329	3.69517E+16
0.304359581	0.001159522	1.44208E+16

0.439057932	2.45795E-05	2.23877E+16
0.498052922	0.000400823	8.80022E+16
0.125153319	0.000211815	2.45392E+16
0.124178132	0.000512009	4.62029E+16
0.22838952	0.000689481	1.91758E+16
0.177216534	0.000957271	2.40935E+16
0.285283431	0.000115633	2.76815E+16
0.345651039	0.001608334	3.84798E+16
0.183315869	0.00224346	3.41608E+16
0.449764635	0.005764699	4.69726E+16
0.176258423	7.0127E-06	2.09626E+16
0.510627904	0.003266526	3.4469E+16
0.378722128	0.002486428	7.22778E+16
0.389284832	0.008427603	1.8704E+16
0.50636215	0.001438861	1.17329E+16
0.349156458	4.59539E-05	3.70973E+16
0.173495595	0.000289811	2.35536E+16
0.187500065	0.001160361	1.61187E+16
0.283220759	0.000323478	1.23992E+16
0.347104636	4.57291E-05	9.17934E+15
0.185481246	8.68574E-05	2.59086E+16
0.117957586	5.88246E-05	3.37672E+16
0.117948977	0.000145056	2.78082E+16
0.266249767	0.000785797	1.79899E+16
0.325818504	0.002683787	3.79575E+16
0.4462866	0.009659384	1.91972E+16
0.20971698	1.9358E-06	2.48133E+16
0.396201302	1.95267E-08	2.13204E+16
0.451074126	1.10445E-06	6.00233E+16
0.253494136	0.000332025	2.63265E+16
0.448664293	0.010353514	5.88398E+16
0.435104989	0.001020623	3.40287E+16
0.720077835	0.028810325	187.1522802
0.341663101	0.005902854	2.38982E+16
0.303783156	0.000217854	2.44906E+16
0.494911157	0.014794241	3.24443E+16
0.33721131	0.006515225	8.46947E+16
0.715922713	0.038437892	3.22475E+17
0.797570922	0.009591053	2.39788E+16
0.737668302	0.000306025	3.10345E+16
0.605823292	0.000165418	2.41257E+16
0.369969861	5.40234E-05	5.2434E+16
0.259122282	0.000326276	1.31651E+16
0.277681044	0.000920945	1.18868E+16
0.199226641	0.000109206	1.1326E+16
0.562411397	0.000224864	2.63236E+16
0.163876892	8.32595E-05	2.28305E+16

0.386174104	3.4806E-06	1.28584E+16
0.191715727	0.000107917	2.60559E+16
0.386435845	3.13374E-06	1.37982E+16
0.504900214	0.002612736	1.20055E+16
0.623521205	0.000586934	1.80454E+17
0.40620663	0.000105822	1.35592E+16
0.533524635	0.012276321	9.11814E+15
0.174234093	4.53489E-05	4.06357E+16
0.212913931	8.60752E-08	5.85415E+16
0.209338547	0.000189443	5.62984E+16
0.354330272	0.000287235	7.72551E+16
0.198718289	2.33515E-05	3.34093E+16
0.449334707	0.003058073	1.23545E+16
0.314871108	4.59699E-05	5.32189E+16
0.415379356	0.000179474	2.60578E+16
0.227308988	1.03726E-05	4.63955E+16
0.545013522	0.001904025	1.28763E+17
0.489684437	0.000457521	2.97582E+16
0.316900375	1.45612E-05	1.05306E+16
0.86456439	0.000778165	1.8561E+16
0.241922812	0.000206966	6.03065E+16
0.243414464	4.03111E-05	1.01062E+16
0.210062114	0.000104136	1.70572E+16
0.128968833	4.01352E-05	1.80467E+16
0.129116341	3.95318E-05	1.54117E+16
0.129651639	3.41379E-05	3.1525E+16
0.216113738	2.88495E-05	2.15872E+17
0.236866726	7.25916E-05	1.52267E+16
0.172034142	6.99688E-05	4.04413E+16
0.360684172	0.002165222	2.17712E+16
0.175760278	9.70506E-05	2.53646E+16
0.546935292	0.000397695	4.32374E+16
0.531368574	9.69014E-05	1.351E+16
0.304897325	6.53109E-05	2.83293E+17
0.499346292	0.000521485	1.57886E+16
0.376804968	0.000212942	1.95621E+16
0.39751627	0.000142893	4.36319E+16
0.729105462	0.001819481	2.89737E+16
0.684611037	0.005323961	1.25897E+16
0.471558739	0.015594752	3.9174E+16
0.762484791	0.006606831	1.16852E+16
0.424678553	0.0013587	2.22097E+16
0.366649524	2.88476E-05	1.07648E+16
0.752280166	0.001377971	4.1734E+16
0.352796655	9.88375E-05	2.17348E+16
0.3676733	8.53551E-05	5.30121E+16
0.419167785	0.001007595	1.67036E+16

0.307389654	0.000131846	1.35964E+16
0.400386305	0.0005675	1.84841E+16
0.633584003	0.007547607	1.51255E+16
0.470792973	0.002436251	1.14894E+16
0.148016132	2.5035E-06	1.59089E+16
0.157915348	1.69969E-06	1.43438E+16
0.158327373	7.17845E-06	1.37052E+16
0.158293056	6.88228E-06	5.49475E+16
0.180490425	6.02058E-06	2.65225E+16
0.180433767	6.2734E-05	2.52827E+16
0.19035591	1.98575E-05	2.64785E+16
0.435231436	0.000654439	3.04542E+16
0.234184146	0.004305082	9.70522E+15
0.505343362	4.30411E-05	2.45381E+16
0.413576782	0.000729291	4.22002E+16
0.35875746	0.009429474	3.57374E+16
0.256066508	0.001668661	3.64483E+16
0.370680427	0.000842318	3.58163E+16
0.530383439	2.80381E-05	1.39194E+16
0.422735729	0.004150016	1.04006E+16
0.25311741	8.78838E-05	2.50328E+16
0.477677378	0.001105051	1.61658E+16
0.456379964	0.000786908	4.37945E+16
0.45561784	0.017236321	1.14004E+17
0.469870076	0.010053919	1.88608E+17
0.590210902	0.001107692	1.37543E+16
0.663440946	0.041875113	1.89507E+16
0.576253739	0.023130932	3.36384E+16
0.514101275	0.011109944	1.15001E+16
0.564822338	0.005227888	4.0867E+16
0.249413042	8.87011E-05	4.41669E+16
0.38904469	0.00156752	2.08983E+16
0.531994108	0.001067514	2.38925E+16
0.480939907	0.006066266	2.05251E+16
0.667559103	0.053171408	3.57078E+16
0.386770938	0.033398633	1.66723E+16
0.346065557	0.014694532	2.20669E+16
0.26230874	0.000314603	6.64088E+16
0.268278892	0.001352068	6.31922E+16
0.244486732	0.000141188	3.14675E+16
0.248232764	3.70718E-06	1.99752E+16
0.305347017	0.000199195	1.6517E+16
0.325193307	0.00396923	2.40645E+16
0.746637347	0.0154517	2.43356E+16
0.541215227	0.002175507	1.92616E+16
0.334643136	0.001396201	1.04969E+17
0.318441126	0.002005863	4.76266E+16

0.322120339	0.0032553	2.60544E+16
0.285323356	0.002974269	1.12414E+17
0.438786386	0.004267593	2.60995E+16
0.362784625	0.002016407	1.37646E+16
0.866071769	0.002107126	3.70112E+17
0.30336092	0.010926844	1.49101E+16
0.327723817	0.001518457	5.59259E+16
0.224853069	0.001523526	1.9765E+16
0.212349077	0.000995507	2.79754E+16
0.248607721	0.001127597	1.97431E+16
0.415751958	0.003803963	2.27095E+16
0.392788935	0.008313397	1.19918E+16
0.337741496	0.000342246	2.56701E+16
0.217781506	0.000920112	2.23511E+16
0.199050148	9.06104E-06	1.03044E+16
0.404136776	0.000475521	9.31623E+15
0.377331284	0.002414423	4.75871E+16
0.233066973	0.007241926	1.81487E+16
0.769133095	0.000148311	1.24808E+16
0.406670894	0.000736575	8.97277E+16
0.383430474	0.000153909	3.46328E+16
0.158986037	0.000128015	3.33364E+16
0.315915658	0.002735358	1.75376E+16
0.425391888	2.58906E-07	1.08423E+16
0.271262201	7.31697E-05	3.75606E+16
0.251304225	1.6558E-06	1.52276E+16
0.257700592	3.8826E-05	3.99329E+16
0.237926664	0.000246513	1.59156E+16
0.183226946	0.000420105	3.94672E+16
0.188819345	0.000261951	3.3523E+16
0.900225411	0.046039417	31485.90165
0.404086085	0.001333369	95.79227163
0.446580691	0.001023129	107.6138998
0.859139924	0.003892462	80.5071334
0.843398508	0.026704197	276.7194146
0.26425973	1.62457E-05	9.89005E+15
0.310972342	0.00024149	3.54589E+16
0.257555755	0.010486612	2.79744E+16
0.226118802	7.96561E-05	4.25125E+16
0.555689393	0.010336365	1.19902E+16
0.306382874	9.18219E-07	1.78182E+16
0.319006614	0.000708501	1.18343E+16
0.294541075	0.004811986	2.88356E+16
0.386289823	0.002113259	4.70093E+16
0.746078782	0.089765769	75.3392789
0.615255833	0.023994629	2.52044E+16
0.262153552	1.74604E-06	2.47486E+16

0.224803714	2.90895E-06	8.40799E+16
0.180244698	0.000572253	4.68842E+16
0.193425728	0.000245786	1.82256E+16
0.253350528	0.000563223	5.2758E+16
0.255195448	0.001406319	2.14195E+16
0.405292954	0.043289908	32.61284194
0.437121441	0.033905168	30.38293178
0.303181527	0.00043548	1.57125E+17
0.402679725	3.06193E-06	1.78583E+16
0.337848477	0.000607971	1.38588E+16
0.714646788	0.000192961	3.76775E+16
0.720832253	0.00260796	1.09702E+17
0.556885854	0.000132251	9.07652E+15
0.900942343	1.655939722	67.3372639
0.92577332	0.629932301	326.3443123
0.617513462	0.006711793	1.24498E+16
0.534576473	0.02548633	666.7091285
0.298729023	0.00018538	10994.12485
0.253254914	0.000155204	4.8364E+16
0.159012178	6.5539E-05	2.64158E+17
0.349446939	3.48918E-06	5.16861E+16
0.144204266	2.42729E-06	8.73082E+16
0.127650068	9.33903E-06	5.62554E+16
0.141139184	6.47659E-06	9.08826E+16
0.164028493	1.37204E-06	3.95346E+16
0.372060883	0.001446991	5.912E+16
0.202014442	2.82716E-05	7.63822E+17
0.219209396	1.27019E-06	1.87253E+17
0.433052511	0.000345047	1.06149E+17
0.537857383	0.002319404	10730.74959
0.433416459	3.78185E-06	12384.16489
0.253849104	1.60392E-05	7.00267E+16
0.453918444	0.000375555	7760.766942
0.326129413	0.000130082	10695.60786
0.616429596	0.004817505	7963.147903
0.255451525	3.45106E-05	4.70726E+16
0.503147964	7.23705E-05	3.0795E+16
0.480281143	1.48039E-05	1.08666E+17
0.408383998	0.000202903	3.84402E+16
0.208769471	5.23781E-07	1.02426E+17
0.435324488	5.34905E-05	6.416E+16
0.201421738	5.66318E-05	5.45447E+16
0.239911482	3.04276E-05	5.26534E+16
0.437002331	0.000133875	11625.88984
0.133975576	2.12442E-05	7.86783E+16
0.114285572	1.20698E-07	8.279E+16
0.154694923	2.02646E-06	2.12612E+17

0.148984404	3.61488E-05	1.94957E+17
0.237717828	9.76333E-05	1.72805E+17
0.147270556	1.30425E-05	2.72262E+18
0.238443176	0.000125938	5.02029E+16
0.129932685	1.5196E-06	2.17481E+17
0.168036102	3.56475E-08	4.04449E+16
0.149381852	5.11656E-05	1.17547E+17
0.138264489	1.72941E-06	9.31044E+16
0.16621794	1.48712E-05	1.14469E+17
0.24297343	0.000244182	9.31319E+16
0.170371125	4.05241E-06	4.81204E+16
0.537850622	0.002032317	308.3386575
0.106526878	1.68414E-05	295.7333866
0.107438731	1.15842E-05	302.0217046
0.114611432	4.31209E-05	309.3159625
0.145046377	0.000120664	310.2620989
0.159861344	0.000191545	308.3476181
0.115619712	4.58297E-05	308.0564093
0.098302498	4.35987E-05	310.5446158
0.298439723	4.96312E-06	288.4954691
0.492968906	0.003077878	314.5539027
0.161737073	0.000350314	316.2962963
0.126810412	0.000238588	318.9130293
0.728781182	0.003515572	434.750594
0.659074673	0.000279171	225.3982508
0.340308378	0.000210047	258.7046223
0.249579271	0.000596754	76.01983242
0.342119272	0.000821426	54.31406883
0.441473686	0.007599004	32.31433868
0.258821584	0.003527364	32.55173721
0.300314637	9.4917E-05	93.90588056
0.597815865	0.00496526	250.8215025
0.426740731	2.39644E-05	85.23918076
0.825952449	0.09887774	91.46080755
0.381434941	0.002560415	54.02426965
0.271403504	0.000784774	52.67800845
0.233555289	0.001582409	48.81568281
0.359698871	0.006537239	49.6203663
0.271053206	0.002840587	151.1948542
0.299934786	0.003486423	151.3325378
0.523032485	0.015445764	125.250385
0.177999571	3.62937E-05	4714.431844
0.178715733	4.00078E-05	875.0790981
0.188582054	8.45587E-06	427.283513
0.155916406	0.00012507	655.2471629
0.139298121	8.74766E-06	1613.198385
0.155325211	1.17258E-05	1391.62272

0.383693848	2.67621E-05	2.63317E+16
0.424859947	0.00010714	2.2424E+16
0.316555775	6.5897E-05	1.15142E+16
0.328669085	1.24103E-05	2.80416E+16
0.402999149	0.000183028	4.04287E+16
0.57210309	3.98454E-06	1287.522738
0.525747568	0.00221281	1380.752516
0.502212591	1.16351E-05	1.71718E+16
0.665531286	0.014419454	360.341836
0.514003683	0.000136607	512.3260701
0.844162477	0.037805062	518.3391535
0.584111824	0.002818765	140.9881435
0.32553255	0.000103636	131.7889246
0.482303972	0.001044645	284.9433357
0.357049187	0.000971707	193.0860728
0.605329656	0.108074882	98.56894925
0.337698195	0.001530616	282.4084151
0.563463077	3.51325E-06	310.4804572
0.316154762	0.000890921	288.8110371
0.150646766	0.000105466	362.6944681
0.149236525	0.001210256	369.2251553
0.162916775	0.000260441	365.5789958
0.18855714	0.000817023	357.2732153
0.169579859	0.000172631	355.3348476
0.999840704	4702.811035	423.6685414
0.113634478	0.000756044	348.5027753
0.113634223	0.000754428	348.5757511
0.305289046	0.005078277	349.921277
0.193612565	0.000677785	344.8535085
0.195488657	0.000939919	2.60649E+16
0.433253805	0.000147896	2.04145E+17
0.722137877	0.003329005	1.76457E+16
0.232161569	0.002185167	2.48558E+16
0.177128497	0.000382719	9.72556E+15
0.245321964	0.002161096	3.6272E+16
0.174865526	0.000965314	2.78225E+16
0.432652256	0.000139721	1.14991E+17
0.118721804	7.60363E-07	2.72708E+16
0.108381343	7.49776E-05	1.1364E+16
0.757526158	0.005112068	3.02272E+16
0.22645012	0.001452587	2.57473E+16
0.194568693	0.001069447	3.30269E+16
0.737231718	0.003387665	2.10016E+16
0.107985594	6.27086E-05	2.0905E+16
0.738372719	0.000171768	1.88262E+17
0.572763859	0.000286086	171.9991899
0.312633702	0.000758728	107.7398066

0.472535719	0.000894861	108.4032858
0.301871992	0.010693088	101.2308504
0.632626285	0.012660179	372.8716289
0.624301859	0.004042316	307.1863976
0.703022394	0.008354811	418.4712014
0.528251025	0.000358219	144.3681697
0.803274482	7.82951E-05	5.06707E+16
0.136151288	0.000118723	3.54905E+16
0.303106738	0.001514544	308.915433
0.387232305	0.002613191	285.639852
0.475877689	0.013157377	265.5647802
0.807499631	8.05183E-05	6.15195E+16
0.181993418	3.78815E-05	2.34512E+16
0.389948774	0.001762924	8.58636E+15
0.215131058	8.41436E-05	1.78761E+16
0.217267406	5.89831E-05	6.87493E+16
0.231098061	7.50417E-06	1.83269E+16
0.623584024	2.60995E-05	3.41118E+16
0.090868583	0.000111791	2.03778E+17
0.145086744	0.000189694	1.40902E+16
0.631000115	0.000433956	4.79739E+16
0.373883606	0.000182652	4.34024E+16
0.104739273	5.60963E-07	1.50512E+16
0.099628392	4.57971E-05	8.01762E+15
0.244370036	0.000144007	2.16136E+16
0.211709142	2.00155E-05	2.78847E+16
0.183432802	2.60288E-05	4.65168E+16
0.125695052	0.0002535	1.29791E+17
0.341434192	0.000142789	1.30845E+16
0.161027483	0.000230899	1.18191E+16
0.520225501	0.000728374	7.76274E+16
0.379794298	0.000458014	1.08111E+16
0.48446665	8.37032E-06	283.5391795
0.277585668	0.000178822	157.7612388
0.364236114	0.000909692	113.5175034
0.392781896	1.98372E-05	106.3934798
0.674113138	0.036358595	107.6799958
0.60816965	0.0128478	174.8348102
0.30331744	1.59847E-05	203.2538988
0.492009542	0.000624526	284.8537654
0.542706224	0.000145169	273.4853843
0.542533063	2.38727E-05	282.6626216
0.60271659	0.001253997	1.26083E+16
0.480654198	9.37852E-05	254.097816
0.147426252	1.35848E-05	437.9255309
0.245985637	5.78934E-05	457.2794636
0.295140282	1.00426E-05	455.1710906

0.222320111	2.55373E-06	451.4091972
0.145261515	1.93096E-05	439.4230801
0.148710358	2.04883E-05	440.6954518
0.626559122	0.012190468	462.1537033
0.557527131	2.67663E-06	378.4018645
0.639010869	0.000208033	2.1617E+16
0.164783262	6.09075E-05	440.2901277
0.426469752	0.001520011	1.75604E+16
0.26109644	0.003355989	1.26748E+16
0.615122357	0.00012878	198.0567317
0.783941527	0.015587102	95.51856257
0.238057417	7.14054E-05	89.28970162
0.528197263	0.000164929	160.1563083
0.380112533	0.005303039	93.60682623
0.289685932	3.42507E-05	94.65091541
0.778765186	0.003808447	176.0865454
0.410718893	0.001103226	90.69077788
0.450402941	0.013638804	180.024978
0.859347371	0.013418945	232.4478489
0.60859966	0.012866896	4.72E+16
0.37799604	0.001053303	1.33509E+16
0.495605468	0.000304595	1.18535E+16
0.462514021	0.022375746	1.45297E+16
0.539161795	0.000777902	6.56301E+16
0.453645438	0.012601787	2.68898E+16
0.665323717	2.28753E-06	2.89757E+16
0.391287398	0.002597892	4.17215E+16
0.491882261	3.6379E-06	1.1084E+16
0.312213241	0.008724618	558.1569321
0.417878246	0.027416697	322.527097
0.973900094	0.345542839	926.3615669
0.328839116	0.000165477	630.852549
0.228860922	0.001032521	529.7774588
0.145245785	0.000419973	501.7913405
0.256539197	0.001722807	635.0588781
0.078173989	0.000156179	636.5958618
0.306252349	0.00179081	607.0309719
0.074743901	0.000305437	585.019046
0.097430789	0.000678419	590.5173486
0.64116915	0.033864485	488.7990425
0.136555087	0.000367697	487.7744555
0.139995016	8.383E-05	644.0170954
0.274192749	0.00206933	632.1775915
0.711929498	0.011508654	633.0243556
0.250285145	0.000762836	646.0194427
0.222106154	1.61273E-05	632.4912936
0.178103138	0.000420882	547.8662398

0.085926303	0.000250112	635.316204
0.376495134	0.002518659	636.1435319
0.27111114	0.003143415	446.3219727
0.137762847	1.49764E-05	562.5480203
0.136331181	1.22464E-05	556.5040949
0.095579104	0.000533702	584.3892388
0.12785693	1.01315E-05	558.8993533
0.758552104	0.001682142	1.51761E+16
0.770615552	0.000967156	1.19333E+16
0.398833373	9.47222E-06	1.26564E+16
0.331415367	0.00053103	2.77971E+16
0.335956454	0.000274994	2.70171E+16
0.272631397	4.45774E-05	7.45105E+16
0.259591605	1.65557E-06	5.76945E+16
0.400212424	4.7286E-06	4.35268E+16
0.797571412	0.019025373	2.29979E+16
0.330360073	0.001444578	4.86543E+17
0.513678621	0.019254713	989.9355215
0.617635365	0.054921708	440.4215208
0.127565907	2.9503E-06	378.9797856
0.234368431	0.003173679	293.7048737
0.317708701	0.000134332	503.3895253
0.118586706	3.97964E-05	376.1708512
0.147600679	3.09501E-05	373.1667085
0.182508206	2.16601E-05	378.9477385
0.168642795	2.12106E-05	381.8722359
0.190532388	4.35338E-05	378.789325
0.179159533	1.08894E-05	371.4934471
0.218877593	0.000124361	375.8121095
0.455466239	0.00050351	409.3586647
0.306727437	6.67794E-05	358.978141
0.702497141	8.92396E-06	302.1657871
0.567000852	4.79317E-05	441.7345506
0.409211618	7.15881E-05	1072.001909
0.556304147	0.000132213	306.8629375
0.465298437	8.0698E-05	554.6172024
0.677245013	0.000348095	1009.722428
0.689877445	0.000365713	1151.626677
0.365309152	0.000312552	704.7848997
0.682423082	0.002448704	830.7928881
0.398154831	0.001595698	1500.320183
0.416252845	0.001044758	759.9373595
0.565910641	0.000129534	2484.999953
0.44581739	6.83098E-05	1060.812982
0.626587105	5.59912E-06	1054.469248
0.183220364	0.000276792	1066.379374
0.167625271	0.000228858	1039.191558

0.168107461	1.63753E-05	1147.801026
0.430733346	0.000389784	1113.773434
0.137756392	0.000164524	831.0231112
0.165699883	0.000378621	829.2865323
0.376420105	9.98253E-05	637.7837292
0.409324404	0.000352305	1.02604E+16
0.085215864	0.000496812	872.9236112
0.118254941	0.000642373	878.388549
0.085418818	0.000492174	874.1057311
0.426983741	3.16594E-05	300.7508846
0.268018782	0.000839576	812.3725832
0.285284744	0.000879665	811.3162042
0.288354464	0.000842925	811.8997397
0.167688282	3.6611E-05	813.7598583
0.137101998	0.000311043	806.1055131
0.495230507	0.003238988	811.587277
0.181275719	1.3885E-05	880.4468105
0.276461102	2.96648E-05	1063.221782
0.227108052	0.000300232	807.9236491
0.367320166	1.32592E-05	589.6726377
0.442496033	2.98946E-05	4.34944E+16
0.280180245	1.11707E-09	2.34095E+16
0.193571795	2.99436E-05	5.38398E+16
0.293425038	1.92577E-05	3.22261E+16
0.254786505	1.44908E-05	1.53099E+16
0.286209995	1.11236E-05	3.43576E+16
0.220246441	7.45458E-07	3.35386E+16
0.207587647	0.000717135	805.7662629
0.185295275	3.04128E-05	805.7502772
0.418367561	0.001753692	6.94773E+16
0.294402022	3.93117E-05	2.12053E+16
0.245926799	0.000129016	1.04046E+16
0.385093188	0.000769077	1.2054E+16
0.374977295	0.000579058	2.98867E+16
0.772877207	0.000411765	916.3330053
0.21757981	5.83521E-05	4.67325E+16
0.257262637	2.24371E-07	1.33035E+16
0.365959012	0.000127136	3.07559E+16
0.396827644	9.68911E-06	1.72967E+16
0.388060549	3.06974E-06	2.28693E+16
0.610319397	0.000520481	3.69693E+16
0.220235196	6.52918E-05	4.83603E+16
0.768384586	0.001558052	7.22296E+15
0.572601915	0.001023393	1.41466E+16
0.411268154	0.000443446	1.3257E+16
0.505907071	0.000425087	1.48042E+16
0.417295912	0.007727559	163.024529

0.642672766	0.001606749	494.4155287
0.642764299	0.001451192	617.0913394
0.235191509	4.02388E-05	1.848E+16
0.205300523	0.000504419	3.01171E+16
0.389012164	0.000219321	2.06238E+16
0.261459256	0.000207543	1.35612E+16
0.286896936	0.000896439	1.7723E+16
0.30561443	0.000398105	1.31266E+16
0.371412001	0.001036892	4.35681E+16
0.533431909	0.000646052	6.5258E+16
0.557315398	0.004000931	5.2568E+16
0.568103364	0.001811335	131.9217981
0.294792945	5.17411E-05	152.7144452
0.303024822	0.000876665	147.2014045
0.203214881	1.80493E-05	3.6169E+16
0.363426161	2.59018E-05	156.4677398
0.383670643	0.000937172	144.9581334
0.313117289	0.00032268	1.96286E+16
0.508040911	3.0674E-06	3.1069E+16
0.70260151	0.006083516	2.96909E+16
0.585792936	1.10174E-08	2.10127E+16
0.456684774	0.000112318	2.49085E+16
0.410061225	0.000561462	4.53084E+16
0.19389141	3.3865E-05	1.2292E+16
0.18722925	5.94748E-05	3.76969E+16
0.460686667	0.005445924	2.62028E+16
0.126230023	6.26253E-05	4.60089E+16
0.122061957	8.72751E-06	3.06938E+16
0.207676891	6.64561E-09	9.55833E+15
0.256465647	2.79553E-07	2.95748E+16
0.378265876	0.000593261	7.59128E+16
0.6807744	0.000837795	8.95273E+15
0.304919088	5.43273E-05	3.63723E+16
0.346258023	8.46767E-05	7.07883E+16
0.346203758	0.004923973	2.07802E+16
0.400472293	0.00149185	4.56261E+16
0.471495813	0.003148172	4.79801E+16
0.239819338	2.19018E-05	4.89032E+16
0.278907098	0.000136592	6.10668E+16
0.53864595	0.000969962	277.5809336
0.199376624	0.00060111	3.40932E+16
0.429875753	2.51597E-05	1.11136E+16
0.488678038	0.000214111	3.42818E+16
0.409811878	0.000109953	1.04718E+16
0.38416317	0.002564709	5.10065E+16
0.593029272	0.003901078	6.03845E+16
0.497683661	0.000342458	2.06351E+16

0.301173926	0.00101724	2.59476E+16
0.221371326	0.000538794	6.18931E+16
0.205857685	0.000219723	2.10126E+16
0.688188236	0.022668271	2.42983E+16
0.398701552	0.000650447	1.04963E+16
0.404970436	1.35392E-05	9.34455E+16
0.364890061	0.005771707	4.35207E+16
0.310897449	2.59273E-05	2.37174E+16
0.354740887	6.35917E-05	5.52295E+16
0.163976528	8.87131E-05	1.70063E+16
0.181035345	7.11989E-06	2.06324E+16
0.518087767	9.85575E-06	5.59275E+16
0.175307994	4.81661E-07	1.313E+16
0.189482408	7.29312E-07	1.54733E+16
0.166865539	1.15519E-05	1.45082E+16
0.435585389	0.001021308	2.29078E+16
0.467428988	0.00014407	4.36527E+16
0.344530185	0.000104006	2.4748E+16
0.551754988	0.022723598	2.19851E+16
0.309714783	0.002337245	1.1777E+16
0.491882311	6.88059E-05	1.8124E+16
0.144469788	2.42997E-05	3.52744E+16
0.247808662	0.000138742	6.7441E+16
0.152443125	3.20085E-05	1.19672E+16
0.343598129	1.49161E-05	1.41716E+16
0.265317168	2.52659E-05	4.12963E+16
0.436006894	0.00438492	1.70285E+16
0.3507436	0.000152452	5.13273E+16
0.391066328	0.000313773	4.64842E+16
0.592480921	0.000457162	1.34654E+16
0.621620593	0.008289289	2.87084E+16
0.346214995	5.91683E-05	1.09411E+16
0.55840837	0.00066192	2.17689E+16
0.64255067	0.014242913	1.4333E+16
0.387070337	0.001872502	2.067E+16
0.511207431	0.00150315	2.32294E+16
0.400134636	0.001159692	1.81907E+16
0.418009569	2.20403E-05	3.21592E+16
0.463944238	0.000231409	9.9232E+16
0.459743218	0.000320437	1.74137E+16
0.335291558	3.81243E-07	9.12726E+16
0.350466789	2.91788E-05	2.02982E+16
0.477509725	2.75163E-05	2.32488E+16
0.441614471	0.000241051	1.54626E+16
0.455552845	0.001283418	1.89382E+16
0.506800126	6.21198E-05	6.89111E+16
0.719634035	0.002411292	2.5121E+16

0.399188716	6.23068E-05	2.10867E+16
0.698472799	0.005514905	1.15291E+16
0.515455863	2.9993E-05	1.69066E+17
0.58348761	0.009325991	3.66048E+16
0.496780726	0.036284992	48.9197872
0.526853449	0.037767027	59.80715762
0.381511462	0.000162507	1087.251953
0.619055186	0.001343738	1096.958868
0.527468958	0.000221532	950.2862085
0.354495583	0.000850494	1246.324456
0.284371818	2.33182E-06	1090.086619
0.220504433	0.000136347	883.0667435
0.252624556	8.12589E-05	882.2667952
0.172802893	1.15653E-05	880.9268842
0.298000859	3.58946E-05	1181.955156
0.508506303	0.000111067	1238.303691
0.474686517	4.86137E-05	1344.260314
0.489666568	0.000496213	109.1838858
0.460420621	0.018011037	81.68495935
0.358703858	0.002269192	107.3177035
0.375299001	0.002994611	101.4335964
0.722550888	0.030331192	80.03169081
0.672383057	0.033924699	534.0141874
0.269842412	0.000456038	306.872402
0.214088865	0.000298763	291.6272751
0.233589761	3.67737E-05	317.488113
0.399506845	0.002301168	392.2863997
0.316097625	0.001719115	392.335198
0.634255756	0.010941205	1814.882983
0.309553339	4.69634E-05	303.3183369
0.411844606	0.000776824	285.1274187
0.274440842	0.00027584	277.3783282
0.253750143	1.55882E-05	194.7560273
0.510923888	0.000336842	196.6518912
0.74546184	0.019050836	262.5504131
0.380355095	1.61535E-05	322.5004937
0.611244228	0.000954428	370.1215313
0.34112829	0.00013072	299.7379913
0.224024443	5.84876E-06	291.9666988
0.27465445	6.80378E-05	313.2206527
0.479955953	1.40413E-05	363.534465
0.429678667	0.000377439	309.1222675
0.675445282	0.018708771	829.8499289
0.479052139	0.000600527	485.3036673
0.225188597	0.000210842	292.4957334
0.408600992	0.00316765	432.1742233
0.473795339	0.000529037	255.253548

0.273829112	1.98847E-05	251.2564639
0.406913166	0.014219499	238.8135181
0.373723218	0.000555025	319.1853837
0.241104692	0.000236628	328.5793294
0.252059888	0.000450959	292.6516357
0.33213159	0.000197788	302.1510264
0.283640259	0.000428678	310.4662087
0.286949144	2.86613E-06	288.6874185
0.232639131	0.000329364	296.2008769
0.132933606	6.28052E-05	290.8103507
0.126269985	0.000127793	291.5239273
0.283797055	0.000306314	296.8279182
0.348267796	0.000381128	2.07124E+16
0.149390998	3.16066E-06	2.46144E+16
0.133179496	2.29927E-06	1.11144E+16
0.529213406	0.005759905	3.33118E+16
0.370570345	5.74974E-05	7.72764E+16
0.3025401	0.001709783	1.72721E+16
0.147425261	5.88067E-06	2.70161E+16
0.134464481	0.00010942	5.1795E+16
0.116153385	3.57004E-06	1.02193E+17
0.171935249	6.91134E-06	1.40033E+16
0.292884988	0.000136647	1.42174E+16
0.15864961	1.63685E-06	1.54531E+16
0.307728998	0.001350095	3.65775E+16
0.274556865	0.000150233	4.1713E+16
0.317372535	2.43734E-05	3.00665E+16
0.164855249	4.90286E-05	2.92323E+16
0.433026301	0.001465261	1.56955E+16
0.295791675	0.000139097	6.07941E+16
0.303328224	0.000330103	9.62557E+15
0.287530532	6.08778E-05	2.68077E+16
0.22831986	1.78544E-05	3.54236E+16
0.668637081	3.74204E-05	1.34788E+16
0.281426244	9.10366E-05	4.48908E+16
0.273680969	0.000658042	1.23122E+16
0.139378908	2.62278E-05	7.25796E+16
0.162994854	2.66827E-06	3.4009E+16
0.152847029	6.2047E-07	2.74758E+16
0.348089828	0.001275372	3.7863E+16
0.141572063	6.03763E-05	2.33076E+16
0.279069758	0.000166148	3.76179E+16
0.131193251	1.206E-05	5.37521E+16
0.479985985	0.004935294	4.48525E+16
0.659670397	0.000130398	2.49403E+16
0.552730169	9.05996E-05	5.13148E+16
0.451657901	0.001467029	5.17704E+16

0.552492166	0.000942229	1.06111E+16
0.450465269	0.00140538	1.40169E+16
0.743466755	0.004998884	3.58658E+16
0.157562827	3.17814E-08	3.47419E+16
0.732451257	0.004361534	6825.112144
0.746957597	0.001595765	7752.221987
0.867817037	0.011493216	2013.877499
0.682724624	0.003310171	229.2418675
0.531942873	0.022300384	394.0120069
0.61100431	0.000722802	2029.283322
0.473327325	0.010912264	295.9783378
0.623774487	0.003079046	434.9763837
0.705680383	0.178604467	1226.46531
0.830613918	0.024832141	444.8441386
0.369357551	0.021671914	352.603736
0.681493088	0.007717023	348.2364164
0.514333426	0.018288896	470.0296424
0.238632555	6.5998E-05	2.48464E+16
0.154020908	3.33088E-05	3.77071E+16
0.143192957	2.79637E-05	1.11838E+16
0.200163139	2.20319E-05	2.09537E+16
0.19658188	8.60142E-05	7.31522E+16
0.279626029	7.7074E-05	4.57726E+16
0.308328337	0.000457681	1.72037E+16
0.184271825	0.000140463	2.35922E+16
0.287196124	1.95363E-05	3.01239E+16
0.24433084	0.000135817	2.15999E+16
0.138831825	7.66909E-05	3.26141E+16
0.198381506	3.02828E-05	3.12656E+16
0.158476021	0.000232805	1.06917E+16
0.216710827	0.000120054	1.34101E+16
0.23027704	0.000124555	1.12876E+16
0.356605498	0.000294398	3.55224E+16
0.15569171	0.000160003	8.56853E+16
0.256190177	0.000291742	2.09625E+16
0.591502741	0.001865665	1.64885E+16
0.298446813	1.11431E-05	2.56175E+16
0.283048926	0.001168639	5.65224E+16
0.674152768	0.045187176	3.99689E+16
0.50780254	8.10581E-07	755.8286106
0.2855422	0.00034362	378.0297672
0.333437893	4.07627E-05	811.256047
0.681124273	0.020160827	6.18133E+16
0.285929192	2.62084E-05	645.1890086
0.455213691	0.007599709	2.60659E+17
0.20237883	0.00121106	1.51511E+16
0.977174796	0.4994742	1.45113E+17

0.596324304	0.017974613	2.88727E+16
0.942696658	0.372641972	275.8869679
0.224879775	2.70997E-05	291.2761216
0.378109987	4.05159E-05	291.4805556
0.321282892	1.33536E-05	310.5668428
0.351590968	0.000972085	308.8480941
0.531047698	7.51823E-05	311.2698652
0.197333652	6.13743E-05	327.8833177
0.516291457	0.002672783	321.0330962
0.213392806	2.73993E-06	326.971286
0.214353923	2.29086E-05	327.199279
0.346454751	0.000482496	329.0613172
0.410164066	0.000299191	328.8991392
0.165180369	5.48474E-05	329.316648
0.152971287	7.95651E-05	329.8477098
0.310481599	0.000265283	330.0342388
0.150155054	2.20493E-05	329.6796076
0.22900373	7.37089E-06	331.0836351
0.406580885	0.000443666	330.9363114
0.373036273	0.006930025	300.3776136
0.445926852	0.01009002	299.7477071
0.489011578	6.03879E-05	347.8166524
0.291432067	0.000130718	339.5679519
0.194933394	1.92817E-05	339.5376817
0.376989953	8.00142E-05	342.8897254
0.203221927	1.06565E-08	343.4514307
0.159230351	9.83875E-06	343.33936
0.382782165	5.47336E-06	343.2439754
