# Supplementary material for: Assessing the environmental factors affecting the sustainability of Aini Falaj system
Source: PLoS One. 2024 May 14;19(5):e0301832. doi: 10.1371/journal.pone.0301832 (PMC11093386; doi:10.1371/journal.pone.0301832)
Supplement: S2 File — Both of and influence Cook’s D values measure the influence of the feature on the estimation of the regression coefficients the dead of Aini Falajs and environmental factors. (PDF) [file pone.0301832.s002.pdf]

Influence	COOKS-D	CND-Number
0.916700369	0.167213902	1091.554729
0.777838352	0.001625223	271.6089225
0.312759566	0.000109461	231.6903796
0.386874981	0.000275878	1575.575331
0.210402666	1.76288E-05	1629.288401
0.326128872	1.9779E-05	1633.931411
0.420887696	0.000505471	1571.042494
0.323169167	0.000211099	1722.160684
0.263275708	0.000325473	1699.38364
0.494274995	0.054904481	1956.177715
0.822260498	0.002916384	749.2354817
0.426841905	0.002118927	302.0169391
0.51843984	0.008741821	255.434774
0.618745439	0.001226499	1234.578762
0.658288042	0.002020183	379.9063973
0.321136158	0.000450444	401.3628462
0.148814106	3.87882E-05	401.716837
0.146136285	2.20606E-05	402.6735076
0.106412032	1.13217E-05	402.7491042
0.155928229	4.79208E-07	403.5267744
0.135319691	2.28956E-07	403.2044601
0.277912231	0.000350276	403.4693917
0.201906745	0.000133613	403.2838283
0.316207759	6.6634E-05	403.1579213
0.224853396	0.000101189	403.2963284
0.924645957	0.009684825	2.62553E+16
0.256578022	0.000125979	8.00509E+16
0.511919466	0.000645444	5.72801E+16
0.160330577	0.000114558	7.06365E+16
0.219937443	0.000131827	4.97158E+16
0.312879769	0.000504819	1.33991E+17
0.53896112	8.48749E-05	1.58585E+16
0.134056325	2.16113E-06	3.45722E+16
0.40914189	0.000264037	1.18204E+16
0.090438096	3.05731E-06	1.12455E+17
0.10817935	1.88924E-06	2.15716E+16
0.132856245	1.46495E-07	4.41353E+16
0.145976907	8.95624E-06	1.28275E+16
0.112635365	8.75432E-07	3.29745E+16
0.101745711	4.07226E-06	3.65241E+16
0.119646201	2.01053E-06	2.84558E+16
0.098520595	9.09437E-07	3.92767E+16
0.103292207	3.83813E-06	3.62705E+16
0.11958471	6.43429E-06	1.2739E+17
0.139657411	1.72355E-07	3.52026E+16
0.141300076	1.22907E-06	5.66408E+16
0.293264895	0.000190626	4.44899E+16
0.134747717	6.25223E-06	2.74382E+17
0.500356106	0.000440815	1.8361E+18
0.387687715	0.000244205	3.15719E+16
0.216539839	3.73175E-06	5.23066E+16
0.145621109	2.12852E-06	5.9964E+16
0.157524371	2.54698E-07	1.19161E+17
0.619939107	0.000149889	3.01597E+16
0.19252648	1.35981E-05	2.64727E+16
0.182370777	1.1764E-05	4.07963E+16
0.263044333	1.39794E-05	2.50552E+16
0.117212307	4.39685E-05	1.83053E+16
0.227310482	1.85589E-05	3.69942E+16
0.519386175	3.18206E-05	2.96098E+16
0.131297209	3.05787E-05	1.16437E+16

0.503028894	0.000105211	5.00453E+16
0.117757096	3.64258E-06	1.69534E+16
0.488296626	0.000294031	2.30039E+16
0.248271474	1.03061E-05	1.03018E+16
0.146290527	0.000243916	4.87749E+16
0.251481398	0.000241723	2.87402E+16
0.368451084	4.45449E-05	3.43336E+16
0.724788636	4.77809E-05	8.72622E+16
0.100958175	3.39905E-05	1.94853E+16
0.166263168	2.71858E-05	6.48632E+16
0.508999536	0.000154282	3.18883E+16
0.782722839	6.78611E-05	1.60872E+16
0.446952734	4.26498E-05	2.55188E+16
0.24834363	1.60521E-06	3.66112E+17
0.420759648	1.33463E-10	6.66132E+16
0.2402807	2.20431E-05	4.32339E+16
0.228111959	8.34737E-05	4.47047E+16
0.160030965	1.67249E-06	3.17584E+16
0.332441206	1.69288E-05	5.84228E+16
0.10751312	3.69423E-07	2.89038E+16
0.123917492	3.84689E-06	3.41728E+16
0.275301157	3.02395E-05	2.11893E+17
0.287226418	3.96214E-05	496.8353434
0.625810725	0.000272434	724.4095165
0.71762605	0.017708923	813.3075344
0.292946164	0.000249424	494.5001842
0.253488625	7.54016E-05	495.3193645
0.337057564	4.19968E-05	233.9460146
0.313849928	0.000193212	233.9049522
0.189541143	1.71991E-05	379.5195348
0.318127393	0.00133531	367.1903032
0.363256233	0.000637337	382.7327122
0.482039265	0.003969402	423.6594785
0.428204253	8.6391E-05	356.5785025
0.348417146	2.53828E-05	333.0419948
0.22637513	1.2328E-05	349.0412221
0.313917479	3.94648E-07	3.40853E+16
0.307473227	1.07502E-05	3.75255E+16
0.338950628	1.47222E-05	1.53495E+16
0.135949641	1.55838E-05	3.25769E+16
0.315268654	0.000303937	4.16794E+16
0.152823302	1.13377E-05	1.08747E+16
0.34430408	4.96002E-05	9.27047E+15
0.484349113	0.000570725	386.6969826
0.423080641	0.000708945	420.8948317
0.49142444	0.007077808	672.436682
0.424233875	0.00106957	679.9075608
0.501554588	2.61038E-06	372.5825017
0.328889244	5.66344E-05	373.0335216
0.329671799	4.61553E-05	375.6108752
0.296625695	3.45721E-05	353.8931233
0.528037943	0.000176592	353.9927722
0.503672963	0.000389437	678.9443276
0.288432154	0.000107083	322.8079061
0.299315901	0.001198397	449.6335185
0.596283855	0.000319022	406.6958488
0.545872608	0.010989089	572.0148679
0.361891047	3.70827E-05	565.0954533
0.298090904	4.0543E-05	566.5567633
0.393057049	3.96997E-05	496.1513635
0.270013479	2.15243E-06	384.6772726
0.149720663	1.30603E-05	406.3885308

0.145902173	1.09686E-05	411.7376365
0.133129209	4.82242E-06	395.6842799
0.126528461	2.86943E-06	414.0105666
0.271204614	0.000136905	405.7384723
0.185956448	7.12539E-06	418.8581664
0.310511595	3.47234E-05	382.1343963
0.1509892	1.05001E-07	419.1564285
0.143846792	1.47074E-05	1.30457E+16
0.451275443	8.76754E-05	2.86889E+16
0.306610751	4.15897E-06	2.67945E+16
0.261706571	1.05676E-05	2.35859E+16
0.275491172	1.3488E-05	2.02266E+16
0.297993405	9.8182E-05	7.47111E+16
0.236573908	0.000398378	2.25213E+18
0.100802569	8.12635E-06	2.58702E+16
0.143357882	0.000169301	1.48096E+18
0.099008818	2.05148E-05	1.74752E+16
0.212829157	0.000243376	2.78121E+16
0.16411186	0.000288049	2.51049E+16
0.404175978	0.002253537	1.606E+16
0.649163975	0.000158055	2.38272E+16
0.31973836	0.000299007	2.33839E+16
0.237064774	4.01817E-06	5.27462E+16
0.811649879	0.019105739	1.86923E+16
0.482355559	0.002024871	1.87122E+16
0.494305472	0.008191342	6.28981E+16
0.309982381	0.000799606	1.32349E+16
0.59967265	0.004482466	9.25542E+15
0.397782117	0.000757323	1.19949E+16
0.367170993	0.000370622	4.11287E+16
0.633964498	0.013370978	5.2828E+16
0.283701322	0.000932458	9.90909E+16
0.231299499	2.42381E-05	5.06644E+17
0.317308343	0.000160232	5.97827E+16
0.322505273	5.82435E-06	1.90888E+16
0.261892875	0.001053311	1.25398E+16
0.510784283	0.000714579	3.15194E+16
0.272687212	0.003650434	3.83661E+16
0.294671548	0.001061347	4.12184E+16
0.573514693	0.002098229	2.27491E+16
0.616101983	0.005591799	1.13918E+16
0.429463502	0.006780253	2.80579E+17
0.471864139	0.001083007	7.93258E+16
0.44511442	0.000362975	3.44223E+16
0.388563663	0.001530342	3.0424E+16
0.699977925	7.3251E-06	196.4415592
0.31632994	0.000259114	126.7045234
0.364487664	0.003095068	135.1074153
0.590483124	0.006275435	145.2302229
0.444742542	0.002438307	108.7992772
0.491500064	0.000103486	92.74964439
0.703470425	9.96378E-05	5.5356E+16
0.652968297	0.003443073	92.52991013
0.72173246	0.017435947	226.2442576
0.376458478	0.018596507	1.32851E+16
0.572906599	0.003074887	2.74954E+16
0.343278824	3.0435E-05	186.3988408
0.530205312	0.003088588	181.7006411
0.534937206	0.000159852	308.8669593
0.230165064	0.000281026	9.29325E+16
0.528215986	0.00106543	4.4294E+16
0.437992988	0.002340804	1.47879E+16

0.262496143	0.000146964	5.08596E+16
0.434782061	8.0552E-07	3.44926E+16
0.378941127	0.000109274	3.32926E+16
0.406550649	0.001908121	3.38795E+16
0.232945225	2.0429E-06	110.9629917
0.302033654	1.48007E-05	111.4036659
0.301837648	0.000107721	171.7932697
0.263695653	0.001287398	131.1286798
0.258214753	0.000192538	2.47215E+16
0.349003605	0.000506389	2.63036E+16
0.411943881	3.53336E-06	1.49565E+16
0.593015447	0.000538233	2.88884E+16
0.686579772	0.046013508	1.92745E+16
0.329611676	0.000104601	3.8883E+16
0.322140331	7.56336E-06	1.90625E+16
0.326164331	2.33263E-06	2.21599E+16
0.361738963	0.000464528	1.48629E+16
0.197382487	3.6957E-05	1.15785E+16
0.180463302	8.88826E-05	2.69017E+16
0.310559115	1.31227E-05	4.33165E+16
0.354166182	4.74149E-05	2.62381E+16
0.450063059	1.30769E-06	3.20761E+16
0.657656541	9.7636E-05	8.82051E+16
0.492038386	5.14827E-05	2.79663E+16
0.740345793	0.010699492	5.68332E+16
0.622274894	1.20177E-05	3.29729E+16
0.291191857	0.003185017	1.28955E+16
0.613976898	0.002782502	1.5018E+16
0.319491472	0.005292696	1.11382E+16
0.221281163	0.000839197	1.01987E+16
0.378875443	0.00382175	1.34462E+16
0.287900183	5.09131E-05	4.38562E+16
0.268279339	0.000716994	2.08379E+16
0.35898831	0.007942718	2.08084E+16
0.322644806	0.001666201	4.38852E+16
0.325020933	0.003950299	1.68437E+16
0.440965268	0.004486296	3.00859E+16
0.240976057	0.000757492	4.51403E+16
0.26150807	0.000738093	3.32113E+16
0.401816989	0.000167736	6.95495E+16
0.300169159	0.004058436	4.7837E+16
0.270432907	0.000200967	3.05334E+16
0.421410902	9.859E-05	3.33854E+16
0.370343772	0.000110127	5.30353E+16
0.350358252	0.002608087	1.23214E+16
0.26599343	0.001144792	1.21559E+17
0.38142499	0.00479083	3.91551E+16
0.30093473	0.010439237	1.89194E+16
0.517440177	0.019306654	2.40738E+16
0.390438541	0.00083853	1.69316E+16
0.229783383	0.002678441	1.68687E+16
0.225858119	0.001441834	2.37733E+16
0.623626438	0.001453878	9.4825E+15
0.51777076	0.0001578	7.49914E+16
0.488097321	0.0017932	1.91454E+16
0.647202472	0.069459367	3.06668E+16
0.690483335	0.255193895	112.0975312
0.252006392	6.85009E-06	6.93682E+16
0.607683818	6.28365E-05	1.9521E+16
0.215989595	4.83059E-05	3.1307E+16
0.890356122	0.307187713	47.44223269
0.455281545	0.00102747	76.98098479

0.419932968	0.000493474	3.44358E+16
0.376524395	1.12766E-05	1.85768E+16
0.263420597	0.001463956	1363.346278
0.218214275	3.14182E-05	1254.359648
0.095723583	9.21605E-05	1272.515846
0.297805244	3.06817E-05	1306.217372
0.141809043	0.000157624	1273.397509
0.137500938	3.2991E-06	1249.655661
0.209004993	1.6539E-06	1246.111864
0.135741593	1.5987E-06	1225.560715
0.099852299	2.04013E-07	1222.096335
0.155612167	6.08075E-06	1218.067117
0.380830777	5.54262E-07	1217.338523
0.226609687	8.83037E-05	1255.861264
0.12119155	5.87448E-05	1253.279271
0.481846323	0.000285045	1121.690298
0.849888527	0.042901816	1480.648471
0.962121817	0.166123457	1032.587585
0.736645661	0.000616655	662.5485067
0.768673238	0.003599052	6.70202E+16
0.795279095	0.011366838	617.2712368
0.794461847	0.001291102	544.2452143
0.710047223	0.000841694	624.4849806
0.673454016	0.002156324	8.73196E+16
0.829247682	0.022761112	608.7972407
0.444352261	0.002199985	2.48038E+16
0.463597139	7.53708E-05	4.5574E+16
0.149483617	1.98663E-05	5.68419E+16
0.1566797	2.19756E-05	4.83347E+16
0.444270901	0.000800812	5.24486E+16
0.257887243	0.000128986	7.26988E+16
0.356748689	0.001216452	3.91406E+16
0.186335631	1.25707E-05	4.42032E+16
0.319840827	0.000402076	6.72415E+16
0.233375158	0.000222904	5.7558E+16
0.236422019	6.51717E-06	9.25472E+16
0.124576074	1.03042E-05	5.31523E+16
0.181303684	2.56445E-05	6.72129E+16
0.419124403	0.000338335	5.3566E+16
0.267516934	0.00022746	3.89459E+16
0.299372517	7.37803E-05	4.59587E+16
0.331203707	1.82542E-06	4.64549E+16
0.235396722	0.000176017	4.01446E+16
0.2210706	4.62973E-07	6.98142E+16
0.165337227	0.000212306	3.981E+16
0.279173756	0.000202268	4.46514E+16
0.280269841	0.0001975	8.24221E+16
0.325741812	0.000365074	7.15212E+16
0.41527463	0.000553611	4.44837E+16
0.140125605	5.9877E-05	3.95179E+16
0.351200992	1.51722E-06	3.61964E+16
0.206489525	0.000425617	4.49566E+16
0.143049811	3.42706E-05	5.35961E+16
0.197657478	0.000108753	4.58722E+16
0.162668006	1.1571E-05	5.04283E+16
0.132524205	3.74399E-05	4.84511E+16
0.138235777	1.79064E-05	4.26185E+16
0.271680578	0.000138444	3.95501E+16
0.187855298	8.68013E-05	5.11358E+16
0.405743226	0.001757516	3.4569E+16
0.197056914	0.000217798	4.68197E+16
0.411684249	0.000344872	6.75335E+16

0.290012135	0.003170453	4.61021E+16
0.438968637	0.001894125	3.95705E+16
0.516273383	0.00071672	1.03565E+17
0.707266503	0.098682239	4.97164E+16
0.88397246	0.045648075	528.0415924
0.22634349	0.000174001	2.52902E+16
0.414345308	8.87131E-06	5.14726E+16
0.311681393	0.000807178	3.08905E+16
0.417290868	0.000169464	5.5894E+16
0.757716462	0.009940486	6.50858E+17
0.756210432	0.000969203	1.7907E+16
0.713694017	0.000838409	4.47883E+16
0.667350847	0.000549406	3.13464E+16
0.29570159	0.00021982	2.87872E+16
0.468556182	0.000412689	1.23608E+16
0.480508728	8.67231E-08	9.10012E+15
0.221796303	1.40586E-05	1.48821E+16
0.538608064	3.66424E-05	2.42794E+16
0.181648468	5.19801E-05	3.10586E+16
0.394441816	5.22209E-05	1.1835E+16
0.276893819	5.1345E-05	3.63462E+16
0.394427239	5.07793E-05	1.25802E+16
0.475168157	8.20844E-05	1.15838E+16
0.475680194	1.65454E-05	7.99854E+16
0.409509039	3.23951E-05	1.35572E+16
0.65667558	0.000243823	7.63097E+15
0.218719686	0.000583195	1.21163E+17
0.251018909	0.000719176	1.99553E+16
0.270536111	2.78785E-08	3.17249E+16
0.269726798	0.00114497	3.82097E+16
0.225205108	0.000345764	3.25807E+16
0.270488922	0.000298198	1.36415E+16
0.5290364	0.002070693	9.37615E+16
0.169175037	0.000517967	2.48641E+16
0.152671752	6.2494E-08	5.14516E+16
0.622846158	0.004815086	5.83272E+16
0.57494066	0.000499181	3.24135E+16
0.193056722	5.83411E-07	9.45377E+15
0.855982477	0.000440406	1.60798E+16
0.217883424	1.08765E-06	2.84889E+16
0.252724317	8.73657E-05	8.64515E+15
0.192813189	2.29822E-05	1.40244E+16
0.107650691	2.69015E-06	1.35973E+16
0.107949131	2.6188E-06	1.3251E+16
0.119513745	1.18853E-05	2.38402E+16
0.159266549	1.20783E-05	5.56395E+16
0.175645657	1.29083E-07	2.02778E+16
0.109652047	1.03322E-05	3.57869E+16
0.2961083	0.001134271	1.77497E+16
0.120700196	7.49591E-06	2.61339E+16
0.578208282	0.004200155	3.03302E+16
0.803295426	0.004228206	1.03787E+16
0.43848938	0.000133654	4.22814E+16
0.413614483	7.96711E-07	1.48159E+16
0.463682886	0.000414566	1.62806E+16
0.53025762	9.84004E-05	3.40267E+16
0.67908147	7.73011E-06	2.42129E+16
0.408984969	2.06661E-06	9.5985E+15
0.310417008	9.48414E-05	5.13868E+16
0.705834716	5.92764E-05	1.26196E+16
0.248208181	5.97137E-05	2.22136E+16
0.281347816	7.47867E-06	9.80783E+15

0.614937993	0.003664771	2.81175E+16
0.287253494	9.52946E-05	3.41976E+16
0.291632263	7.51212E-05	3.34737E+16
0.469195706	0.000116983	1.40471E+16
0.274978049	0.000125661	1.25828E+16
0.314244508	0.000385252	2.32519E+16
0.484287628	0.001359474	1.17676E+16
0.459024437	9.23676E-07	1.34575E+16
0.214025858	0.0006583	1.52284E+16
0.154071676	3.10817E-06	1.41349E+16
0.160881508	2.24228E-06	1.36682E+16
0.160826923	2.03262E-06	5.98421E+16
0.234232884	0.000118938	2.93979E+16
0.194236641	2.17556E-05	2.27421E+16
0.176417117	0.000142958	3.17221E+16
0.345011216	4.43403E-06	2.5865E+16
0.467066739	5.52788E-07	1.14827E+16
0.465193493	0.000432144	2.06678E+16
0.48247087	9.2879E-06	2.09903E+17
0.534751992	0.001497543	2.47186E+16
0.291899118	0.008635093	1.85557E+16
0.492471566	0.001911428	1.58119E+17
0.539072047	0.000686398	1.18129E+16
0.370738014	0.00011668	4.83595E+16
0.335307895	2.52076E-06	2.81926E+16
0.306048618	0.000113691	5.42642E+16
0.491285	0.000351442	3.57092E+16
0.598571862	0.002433283	2.13074E+17
0.454640962	0.00151845	7.52087E+16
0.715893637	0.057393446	1.28698E+16
0.477558811	0.004802922	1.62179E+16
0.387746829	0.000104599	3.08208E+16
0.251175146	0.001631521	1.28953E+16
0.243529756	0.001909519	3.80571E+16
0.500555266	0.001385379	4.79918E+16
0.195472742	1.77247E-06	7.24571E+16
0.446290195	0.014071522	2.0602E+16
0.529112293	0.005248487	1.56928E+16
0.54717079	7.78062E-05	1.15409E+16
0.481117338	0.002222079	1.59845E+16
0.385217953	0.00135427	2.26574E+16
0.319356483	1.66996E-05	3.40587E+16
0.286653721	0.00017441	7.03474E+16
0.270715569	0.001785947	1.89726E+16
0.30791684	5.39033E-05	2.94019E+16
0.43381626	5.44757E-07	2.01647E+16
0.381307723	0.010377877	2.98668E+16
0.868061959	0.001041535	4.04558E+16
0.445171198	0.001711881	1.95442E+16
0.253203848	0.000130033	8.18262E+16
0.34708306	0.00070641	2.82889E+16
0.411344035	7.29797E-05	4.42074E+16
0.204331608	0.0013845	2.76293E+16
0.303484845	0.000865091	3.04491E+16
0.33613474	0.00105183	1.70601E+16
0.840285471	0.01596959	1.66267E+17
0.241815746	0.000232259	2.11159E+16
0.346698486	0.00093664	1.00563E+17
0.266378152	0.001212249	2.16075E+16
0.328584118	0.003691304	2.16179E+16
0.254328351	9.50062E-06	3.22269E+16
0.292449439	3.67432E-06	2.74161E+16

dried

0.361557387	0.001442235	2.36876E+16
0.310201617	0.000193974	1.47796E+16
0.288318117	7.03485E-05	1.54564E+16
0.300080293	0.00085415	1.64501E+16
0.563720861	2.97299E-05	1.03049E+16
0.390658467	0.00223164	2.49042E+16
0.221977502	0.00042643	3.25619E+16
0.709142039	0.012450838	1.21448E+16
0.550393178	0.00104758	5.7458E+16
0.247948377	2.23548E-05	3.0485E+16
0.230255597	1.8733E-05	2.166E+16
0.188361849	0.000582178	2.02512E+16
0.231184996	0.000139872	1.12339E+16
0.20590454	6.69106E-05	2.60803E+16
0.168542976	0.00011537	8.96671E+15
0.248563324	0.000103501	1.93605E+16
0.18698588	0.000148385	1.11519E+16
0.290848867	0.000227834	2.17074E+16
0.162767492	0.000635813	2.3381E+16
0.930074059	0.076489531	29696.87162
0.436238352	0.000513604	75.54368726
0.424508131	0.001707323	76.39355383
0.662342326	0.001875808	71.98228826
0.86109883	0.001412799	195.8925803
0.195349927	8.70217E-05	1.00171E+16
0.979771284	0.00424775	4.0785E+16
0.2127636	8.42223E-05	2.16969E+16
0.228757295	0.000229961	3.27763E+16
0.521339859	0.005659064	9.92899E+15
0.245313049	0.002655804	1.6713E+16
0.431645541	0.000642774	1.74666E+16
0.275675879	0.001764038	5.6135E+16
0.267775277	0.000309291	1.21316E+16
0.81076645	0.012254806	81.68141714
0.405873241	0.00015121	7.64145E+16
0.252004969	0.000355335	3.48885E+16
0.241618021	0.00041232	3.88819E+16
0.200125686	8.16841E-06	1.94988E+16
0.2264696	3.65141E-09	1.69948E+16
0.259408212	0.000768066	5.65E+16
0.138089336	0.000355534	1.83306E+16
0.447175905	0.000758957	35.83945175
0.507127354	0.00119453	32.94273726
0.329592107	3.74991E-05	5.76469E+16
0.381789838	0.000194834	1.4234E+16
0.334546971	7.26976E-06	1.53629E+16
0.413976028	6.81453E-06	2.98024E+16
0.423256881	1.73835E-06	5.95944E+16
0.585735507	0.001866675	9.10614E+15
0.896290581	0.159900127	76.34521936
0.844102029	0.02732752	600.4463658
0.598715112	0.000180631	2.60434E+16
0.502931649	0.008135106	667.2706998
0.237149552	0.000184598	2.5289E+16
0.384769909	5.4993E-05	7.0191E+16
0.153168746	1.28073E-06	4.93377E+16
0.262639964	3.04496E-05	3.27638E+16
0.158851351	5.23771E-05	4.08901E+17
0.12465042	1.84542E-05	2.24114E+17
0.139158598	2.03369E-05	6.6267E+16
0.139325723	1.37582E-06	5.41535E+16
0.40210372	0.000194318	6.22954E+16

0.225511842	6.43572E-10	9.0446E+16
0.148907575	1.58054E-05	6.73393E+16
0.406519765	4.44454E-05	2.0988E+17
0.516010141	0.000812397	1.80357E+16
0.250773416	5.78361E-05	2.28404E+16
0.268411704	1.1127E-05	1.12205E+17
0.439538716	0.002361615	2.91664E+16
0.307931332	0.000391254	2.38826E+16
0.533161915	0.021559209	3.20386E+16
0.410149603	0.000470457	7.94346E+16
0.482701067	0.0045841	6.21375E+16
0.290703193	0.000459348	7.8649E+16
0.471443423	0.001818771	1.743E+17
0.210414302	5.06371E-06	8.11922E+16
0.260303548	0.000246529	2.36439E+17
0.258147719	1.17229E-10	7.24847E+16
0.311894966	0.000156865	3.87599E+17
0.235782929	0.000438839	2.43108E+16
0.216165679	1.82853E-06	2.96948E+17
0.159964474	4.10774E-05	1.22706E+17
0.145655083	4.12497E-05	7.77698E+17
0.255457655	0.000149763	6.8982E+16
0.222931394	0.000185351	1.10876E+17
0.153275075	1.59977E-05	5.24631E+16
0.34244913	0.000293889	5.58187E+16
0.136339113	6.19913E-07	8.15756E+17
0.166015611	1.99261E-05	6.01017E+16
0.140045258	5.81891E-06	1.18752E+18
0.112180729	1.60802E-05	7.79796E+16
0.135623492	2.90871E-05	6.40874E+17
0.099274384	7.23819E-06	1.13117E+17
0.175185411	5.5436E-05	4.9793E+16
0.484270626	6.01509E-06	267.5407506
0.134574742	4.48352E-06	272.9895761
0.123266715	1.04147E-05	283.8004735
0.103595699	4.21404E-06	296.5274205
0.186518359	6.88359E-08	297.9431379
0.273579567	1.00249E-06	295.1147758
0.114519891	3.5991E-06	294.6725548
0.104008044	3.47851E-06	298.178465
0.186805987	2.21123E-06	259.0596039
0.170333401	9.39135E-06	303.1061616
0.426428882	3.00492E-05	305.0698478
0.403817816	0.00010564	307.760871
0.728141297	0.008799398	400.0787341
0.542085209	0.002384396	177.6202666
0.345332328	0.000984119	230.5845302
0.614264524	5.6513E-06	73.91100659
0.300527173	0.000939723	57.26499442
0.551118036	0.036239534	25.86150215
0.464786165	0.000370019	26.43556515
0.69392112	1.20807E-06	86.31915355
0.522703105	4.79717E-06	222.0828639
0.380909188	0.00188887	63.04038085
0.829082103	0.000316424	103.6080709
0.512178694	0.001086205	51.47632759
0.414515219	0.000534279	50.49793268
0.374179641	1.87646E-05	47.697011
0.672205941	0.001193231	48.26033191
0.385459638	0.003697878	105.4954113
0.625085985	0.003506623	105.7967129
0.48274754	0.00374167	62.36514907

0.201539376	0.000197543	4481.772036
0.149699188	0.00012156	837.85597
0.224918506	1.2629E-05	409.4509449
0.306445779	0.000374267	628.8745055
0.227197442	3.27339E-05	1554.812056
0.137712157	0.000127739	1340.417834
0.316460971	0.001713358	2.0979E+16
0.342802637	0.000135493	2.7711E+16
0.261911678	1.8357E-06	1.10752E+16
0.570357402	0.00036708	9.26536E+16
0.570986559	3.07353E-05	1.53382E+16
0.5046553	0.000166948	1221.922518
0.495168899	0.000949405	1314.272944
0.486921881	0.000273129	2.25737E+16
0.389402987	0.000847567	183.4655722
0.614880477	0.012568917	185.5409769
0.858400282	0.121922849	399.4921989
0.834327354	0.03333685	122.0468762
0.534011264	0.000722041	79.81862118
0.419807395	0.000305024	176.839652
0.565239064	0.005946614	147.1453827
0.525629531	0.009319109	53.09393533
0.397676099	2.34626E-05	251.2284749
0.612597838	0.000287825	208.2910062
0.335358355	0.000189101	243.7968934
0.172307355	0.000149498	218.3710707
0.216132981	0.00070639	224.9034396
0.134045242	4.77246E-05	221.2356992
0.323859937	0.001989302	212.0671985
0.14803936	8.13007E-05	210.4557042
0.999731835	422.3548194	317.5600055
0.168540587	0.000206923	204.1105079
0.168540311	0.000206583	204.1811213
0.170644386	2.25657E-05	206.1278222
0.210055589	7.32369E-05	206.6162449
0.185650397	0.000104814	2.07127E+16
0.477418906	5.43895E-06	2.19686E+17
0.885401989	2.24843E-05	1.82041E+16
0.146742132	0.000509174	1.99202E+16
0.225165029	0.000219066	1.25787E+16
0.279851527	0.001241257	3.28843E+16
0.250045815	0.000141837	2.62428E+16
0.477461606	7.26853E-06	1.11336E+17
0.25641205	7.94586E-05	3.76663E+16
0.180846344	4.05313E-05	1.35799E+16
0.181507277	3.3776E-06	2.4391E+16
0.253669723	2.36616E-05	2.70601E+16
0.149184306	4.83053E-06	2.35421E+16
0.56683998	0.000103179	1.99929E+16
0.180045158	4.71878E-05	2.31213E+16
0.534603361	6.46327E-06	1.02212E+17
0.63716963	0.00189262	162.9150481
0.364530944	0.001175485	97.32668964
0.456602593	8.18337E-05	96.91121405
0.506359728	0.006211357	117.7638107
0.640290595	0.002653603	213.5796934
0.502313706	0.007658705	213.8036177
0.771454619	0.014242035	610.5899713
0.72586519	0.000482263	227.2749943
0.687874457	0.000537226	1.6634E+17
0.11160029	7.41059E-05	2.31103E+16
0.420251808	0.003797198	235.9821563

0.644095676	0.000656299	207.756341
0.495271793	0.000122761	175.9648832
0.681215814	0.000313724	8.54242E+16
0.164629573	1.05483E-06	1.45957E+16
0.342093114	4.06745E-05	8.02977E+15
0.175755222	8.85495E-06	1.72469E+16
0.191929203	3.61277E-06	4.26535E+16
0.133831427	2.74292E-06	1.57269E+16
0.561750772	0.000205473	5.85043E+16
0.100143302	3.30374E-05	2.99522E+16
0.128362744	1.58139E-06	1.84959E+16
0.477914657	0.00105976	1.62497E+16
0.494569872	5.59148E-09	4.86474E+16
0.092842034	1.12418E-05	1.94211E+16
0.125205539	6.50966E-05	9.08597E+15
0.169051416	9.64703E-05	2.1934E+16
0.173469373	4.90355E-05	5.534E+16
0.10189441	7.66108E-06	3.22205E+16
0.085447485	4.46688E-05	2.46121E+16
0.504461492	8.71007E-05	1.68934E+16
0.136131619	5.06513E-05	9.02365E+15
0.452364635	0.01544568	6.32522E+16
0.210256165	2.15572E-05	1.24725E+16
0.536474914	3.80698E-05	274.4869109
0.24226147	0.000379154	135.5858482
0.256496006	0.000240696	93.27348505
0.266094433	0.0002458	85.79654594
0.579574614	2.41761E-06	96.00918744
0.398060849	0.002694992	164.1289205
0.350645445	0.000152051	191.3993921
0.273183844	1.22497E-05	275.9520377
0.462253683	0.00024274	263.3524408
0.278516065	6.07857E-05	273.3982155
0.686595451	2.91007E-06	2.1648E+16
0.446301948	3.10289E-05	260.5046191
0.206767675	1.39398E-05	411.5765664
0.216659841	4.56463E-06	434.8576308
0.263887107	6.97626E-05	431.734868
0.123119381	1.07901E-05	427.0011166
0.230636315	3.30355E-06	414.0149716
0.219216106	3.69022E-06	415.8816343
0.430091661	0.001368479	442.9195618
0.35714935	3.32499E-07	367.4432324
0.550135273	0.001144949	4.5124E+16
0.273995243	3.76486E-06	415.4149774
0.832175885	0.017420154	1.30808E+16
0.31803451	0.000652772	1.4054E+16
0.534751657	0.000646008	211.7756682
0.816300621	0.001485483	88.24140456
0.255010484	0.000596184	74.22737745
0.466838754	0.001465562	168.6657002
0.540890831	0.001493258	81.14716213
0.776182133	0.008472239	83.40560859
0.773250314	0.004646273	109.1279642
0.340787915	0.00087113	75.25842894
0.466744899	0.000193783	248.8698819
0.86138464	0.000901669	294.5442172
0.43300978	0.001381024	4.90193E+16
0.450100429	0.000335811	1.08537E+16
0.276336844	0.002672432	1.03199E+16
0.4715469	0.003353536	1.65978E+16
0.312736037	0.004286717	4.13775E+16

0.671966275	0.001679842	4.40052E+16
0.640113322	0.003468166	2.93178E+16
0.548292182	0.000160613	8.4525E+16
0.367655226	1.88554E-07	1.24385E+16
0.349419769	0.000583276	302.9149941
0.472542318	0.001502168	197.254988
0.963083517	0.439855478	279.3557499
0.203294556	0.00034766	279.1297042
0.166050981	8.22299E-05	268.758819
0.181032212	4.33615E-06	260.5662302
0.187767556	0.000229307	296.0543162
0.170095258	0.000211514	294.6469769
0.140246776	7.28465E-05	289.7783588
0.108008695	2.81366E-06	283.6925701
0.155068557	0.00011255	284.7337348
0.560699521	0.001311795	312.735689
0.110279338	5.73627E-05	256.291678
0.289182484	9.21106E-05	265.1904939
0.427394402	0.004634334	274.6666761
0.261497867	0.001516284	281.0831167
0.448179763	3.67601E-06	264.3338747
0.435260571	0.00352488	274.1908031
0.190016851	6.28905E-05	273.8175186
0.218204716	3.02647E-06	294.6601971
0.261154615	0.000203165	293.2474103
0.527398847	7.00886E-06	242.4603353
0.108557531	2.85752E-05	278.0928171
0.122063913	9.30722E-06	276.4995577
0.09265839	6.88282E-05	283.7950004
0.106237399	1.95494E-09	277.0457567
0.837610233	0.006269809	1.41156E+16
0.840364066	0.024457723	1.95375E+16
0.352683495	8.29835E-05	2.11641E+16
0.412500075	6.57761E-05	2.12152E+16
0.241936345	0.000752018	2.53798E+16
0.332105919	0.001234711	6.62559E+16
0.235206932	0.000408421	4.5135E+16
0.345949326	0.000102192	9.17322E+17
0.720470202	0.134959398	3.41883E+16
0.339893576	0.002655722	2.1506E+16
0.764866248	0.071661815	1057.122655
0.592786468	0.128398522	389.1966266
0.210423455	7.46892E-05	313.4278059
0.32066839	0.00218692	339.7212792
0.331078241	0.001009805	652.0738106
0.131237462	0.000186811	310.886268
0.1521151	0.000472305	309.9130474
0.185198714	5.4908E-06	313.2998525
0.626716167	0.00747983	316.2123471
0.120914139	1.59369E-05	313.4694432
0.269117059	0.001349869	307.1594697
0.227297591	0.000600327	311.2957319
0.362214373	8.62651E-05	537.5014704
0.319011926	0.000953712	535.6473674
0.578441269	0.006861765	420.9462994
0.499928961	0.000532745	659.8205876
0.184833916	6.69488E-05	942.8553858
0.477253133	0.006924879	249.9469839
0.387663998	0.000772072	503.6490456
0.740666534	0.12289321	891.0139256
0.872674049	0.000841848	1109.611024
0.325087241	0.011267095	575.1593505

dried

0.420763718	0.003575878	759.4135461
0.473577533	0.006708166	430.9064872
0.29231251	0.002297941	530.0516921
0.446689075	0.005189771	1612.360555
0.360151164	0.003268388	1132.792646
0.357617488	0.006179615	980.2564586
0.247133616	0.000692781	935.3024398
0.210910441	0.000175542	898.5080219
0.175325353	8.54764E-05	1107.514609
0.377066704	0.011278242	1240.496032
0.159589566	0.004475025	697.0865988
0.204087665	0.005355855	695.7939441
0.412845599	0.031952441	2.30818E+16
0.315701848	8.91396E-05	5.61136E+16
0.1407338	0.005315969	723.8504426
0.170293552	0.00526863	728.1187227
0.141157738	0.005298137	724.7982576
0.313119187	1.29675E-06	230.8454608
0.465725015	0.003599021	766.3175767
0.367793442	3.31654E-05	763.8219665
0.378251957	7.71051E-06	765.2715791
0.212611523	0.001476576	770.9428416
0.168986643	0.001396789	738.7098572
0.205605923	0.009426269	750.8869522
0.146789042	3.16349E-06	842.1002346
0.227641877	0.000130845	906.6473493
0.134199297	0.001142166	721.1358374
0.433929905	0.000626952	3.45631E+16
0.363074235	7.5197E-08	6.91092E+16
0.118008228	6.54387E-05	4.4861E+16
0.14515839	5.26923E-06	9.38645E+17
0.231696982	0.000355761	7.21723E+16
0.151474783	1.08373E-05	4.79791E+16
0.248412079	1.0791E-07	8.12912E+16
0.240241258	0.000535371	7.11755E+16
0.161813631	0.001185832	737.1991632
0.103383913	4.9224E-05	694.9175098
0.418616947	0.000242707	6.71533E+16
0.305151454	0.000202096	4.66171E+16
0.313540406	0.001259923	6.89402E+16
0.381970312	2.59048E-05	4.13831E+18
0.35811885	6.48992E-05	3.91465E+16
0.794368261	0.022372658	1969.846373
0.229069469	1.92979E-06	7.62846E+16
0.305822458	5.27387E-05	1.21392E+17
0.34733506	0.000614941	5.97847E+16
0.306967981	0.0009489	3.98571E+16
0.257929218	0.000419404	2.53421E+16
0.229651504	4.72511E-07	3.50137E+16
0.117301484	6.128E-05	3.89222E+16
0.412489507	0.001030755	7.64596E+15
0.344529169	0.009099239	1.48291E+16
0.347273897	0.000950941	3.20162E+16
0.359488861	7.35657E-05	6.24405E+16
0.42708731	0.079693811	159.3254353
0.730458593	0.024055818	680.4099121
0.74075729	0.022642961	737.1713798
0.248956781	8.46805E-05	1.83273E+16
0.141458532	1.22758E-05	2.37849E+16
0.155912047	0.000116526	1.43018E+16
0.244514727	6.20616E-07	1.25353E+16
0.217774947	0.000246411	1.66973E+16

0.205547154	3.41014E-05	1.55576E+16
0.230185538	1.59776E-05	2.7401E+16
0.558238898	0.020006842	1.32872E+17
0.401821532	0.006273088	3.9255E+16
0.425249354	0.015333635	130.1670261
0.196087833	0.000423352	148.4395031
0.280621111	0.000326158	143.4123777
0.135182319	2.92121E-05	1.29255E+16
0.458692845	0.006188878	170.9811551
0.857125712	0.049035614	156.5828685
0.261669828	0.000563704	1.48544E+17
0.465184038	0.000244239	3.65448E+16
0.89112649	0.078343471	5.00169E+16
0.565574806	0.009197473	3.01628E+16
0.562016587	0.014781778	1.31003E+17
0.372177749	0.000302894	3.42624E+16
0.226063423	0.000427933	1.32725E+16
0.202414994	0.000714192	3.57869E+16
0.312899154	0.000721148	3.57614E+16
0.165615	0.000500173	3.21074E+16
0.183299941	0.000219359	2.38506E+16
0.167977427	9.91413E-05	8.50191E+15
0.126537647	0.000124593	2.37884E+16
0.61434172	0.007408027	1.86082E+17
0.603211963	0.014108296	8.2023E+15
0.352045218	6.75562E-05	6.60997E+16
0.454093347	0.004731962	7.46702E+16
0.427000789	0.025007066	1.95225E+16
0.223187133	0.000236168	7.33095E+16
0.351440716	0.000401512	2.74459E+16
0.510933275	0.000101186	4.46218E+16
0.514802365	1.25996E-06	3.7802E+16
0.605410296	0.005632074	133.1908862
0.315194266	0.002168652	3.13174E+16
0.299016165	0.000585219	1.05068E+16
0.286083422	5.32098E-05	3.2341E+16
0.561356426	0.001046011	8.33115E+15
0.277901707	0.006104267	3.03758E+16
0.504022215	0.001623451	6.8921E+16
0.475151306	0.000945634	5.52775E+16
0.283751012	0.005322552	2.44528E+16
0.309670646	0.00485085	8.685E+16
0.507770821	0.006723416	1.67901E+16
0.417926868	0.027911011	3.15713E+16
0.302141995	0.001927623	1.71145E+16
0.395230202	0.009064311	3.05671E+16
0.378713599	0.037320504	3.62908E+16
0.302194861	6.85009E-06	2.52039E+16
0.338164181	0.000252786	4.50159E+16
0.33791932	0.000936714	1.4245E+16
0.190290415	1.603E-07	1.91428E+16
0.583796187	0.002289438	2.98649E+17
0.268724776	0.000147551	1.17391E+16
0.229456631	5.14382E-05	1.83857E+16
0.187945044	1.38032E-05	2.23554E+16
0.351209671	4.09422E-06	2.62984E+16
0.269781995	0.000605579	9.78758E+16
0.289052556	0.000256312	2.42794E+16
0.511438069	0.001577117	2.09816E+16
0.353763737	0.010364676	1.29343E+16
0.502355018	0.049535534	1.46407E+16
0.179003745	0.000173785	2.3852E+16

0.240879174	0.000118464	4.29234E+16
0.215486608	0.000177593	1.18572E+16
0.342434411	0.003438398	1.70946E+16
0.200266605	0.002225857	2.83396E+16
0.567712963	0.002117763	1.70244E+16
0.268977504	0.000961782	2.32587E+16
0.287728116	0.001076671	3.66567E+16
0.303860012	0.003893028	1.12409E+16
0.774969428	0.000213854	2.03199E+16
0.314435745	4.73315E-05	1.05806E+16
0.648845147	0.082685721	2.8627E+16
0.61373678	0.023395596	1.25259E+16
0.31080852	0.00032149	4.2081E+16
0.261066768	0.00102998	1.56139E+16
0.412790762	0.003458251	1.41908E+16
0.403478596	0.017631501	3.37706E+16
0.318525945	0.001405239	6.51569E+16
0.762565706	0.025939251	1.40064E+16
0.412475117	0.000355904	6.02193E+16
0.489525432	0.00034682	2.09309E+16
0.400768496	0.000701249	1.93895E+16
0.421448894	0.002677025	2.08543E+16
0.356631565	5.48743E-05	1.62071E+16
0.35019316	0.002057565	3.04558E+16
0.726653839	0.023372448	2.3095E+16
0.261336924	1.13787E-05	3.40273E+16
0.632917183	0.015172434	6.31519E+16
0.324948421	8.38905E-05	2.90459E+16
0.536721043	8.91868E-06	4.94897E+16
0.575101959	0.005785277	39.51012665
0.707112673	4.76657E-05	51.26957509
0.241255309	2.93118E-05	4755.302115
0.264224696	0.000189807	971.5916331
0.540221308	0.000570682	801.6194859
0.313336401	0.00060193	1662.137116
0.265149367	4.01168E-05	4662.045578
0.281310144	5.30431E-05	259.253328
0.294205099	1.1315E-05	261.0362379
0.236844631	2.20094E-06	256.6920616
0.436779283	5.38011E-07	4374.851919
0.423017199	0.00197384	1603.193352
0.636905999	0.016336744	4988.719526
0.589129787	0.000401425	113.1938373
0.50369225	0.005982069	68.74241021
0.48499681	0.000440933	110.6477193
0.399097864	0.001243964	101.6648332
0.760995478	0.019056596	76.55351047
0.659338335	0.008095166	349.8242361
0.338987239	0.000181588	236.3002481
0.212958607	0.000318102	189.9477833
0.225078529	0.000370392	244.3047444
0.33034648	0.00547173	975.0592107
0.313379623	0.008137083	979.3174554
0.455722483	0.004185398	10218.57259
0.704721505	0.009304843	281.324191
0.316922477	0.000956827	267.7951377
0.295989371	0.000375098	254.866663
0.218705963	3.54506E-05	174.8330129
0.383299969	0.000660039	176.502703
0.769793412	0.074290649	226.2466182
0.541387171	0.000391033	467.4543725
0.621355012	0.000507812	342.8019012

dried

0.550660623	0.005436451	385.1229008
0.276132705	0.00014716	362.5699531
0.154413431	0.000216037	436.717239
0.167690336	6.35639E-05	671.172878
0.231784433	0.003370383	421.1323103
0.675517305	0.001558021	462.1216003
0.573356866	0.007325631	202.3035408
0.118388046	4.53639E-05	269.6041083
0.854318577	0.016630947	193.5844679
0.605329864	0.000119974	220.8120469
0.393914869	0.001634207	219.1804976
0.335383732	0.004598088	223.9886416
0.651545303	0.00131747	306.4502274
0.244979353	0.000201492	295.9070961
0.156249584	0.000292276	264.485781
0.15379961	1.91366E-05	265.7047128
0.200380348	1.22511E-05	270.4199659
0.324263916	2.72206E-05	256.1207488
0.244393522	7.86369E-05	268.9449767
0.152566867	2.34277E-05	262.0910199
0.155337685	8.33796E-06	263.0305102
0.258249339	7.36256E-05	269.5584834
0.285301934	0.000324238	1.96774E+16
0.172586055	0.000405891	3.09782E+16
0.140631797	0.000174617	9.75586E+15
0.535181516	0.000184948	5.80012E+16
0.325618305	2.20392E-05	1.05197E+17
0.348453289	0.00706795	1.5612E+16
0.154842974	1.25961E-07	2.05045E+16
0.17411047	6.46507E-07	1.4651E+17
0.14820027	2.47679E-07	6.60962E+16
0.143783644	9.59299E-06	1.46867E+16
0.139724745	3.69027E-05	1.27426E+16
0.10551027	4.63556E-06	1.52349E+16
0.239230216	6.35032E-05	1.80178E+16
0.141154907	2.95642E-05	1.6978E+16
0.290164797	0.000301938	2.64223E+16
0.192462393	1.88665E-05	2.34474E+16
0.409186996	0.000562692	2.19697E+16
0.342047852	0.001202137	7.93796E+16
0.331762805	0.00029717	8.21877E+15
0.198875929	0.000129391	2.23062E+16
0.234549278	0.000286937	3.6315E+16
0.678328153	0.026186508	1.1457E+16
0.265463105	0.000266195	2.58758E+16
0.331282354	0.006976047	1.15216E+16
0.133467441	0.000414866	2.77496E+16
0.198434752	0.000588165	7.87446E+16
0.208134395	0.000660508	7.15504E+16
0.324117977	0.006140217	4.03804E+17
0.161113015	0.000148925	1.90654E+16
0.257005439	0.000619286	5.36724E+16
0.126105871	0.000184078	6.51189E+16
0.508891835	2.81691E-05	4.54743E+16
0.552117333	0.000230316	1.82964E+16
0.410268054	0.00010933	5.52007E+16
0.359585688	9.77104E-06	2.18957E+17
0.402889012	0.000339407	1.01631E+16
0.287696944	0.000218206	1.37654E+16
0.850402714	0.000165548	6.88912E+16
0.12011972	3.40844E-06	5.3939E+16
0.784706683	0.001633112	5646.756348

0.811109769	0.030564214	5910.703924
0.882184199	0.01275102	2261.182108
0.615124447	0.004528122	180.559831
0.511134474	0.001550956	453.3871755
0.557160747	0.019954919	1216.205048
0.195570019	0.004263602	276.6418176
0.555983175	0.001154675	503.3151943
0.741364287	0.428184137	1016.106742
0.818567834	0.005825773	368.9518583
0.372424106	0.000928968	294.4709209
0.731384705	0.000353076	574.306837
0.465103035	0.022526774	682.305459
0.274063484	1.30228E-06	3.44133E+16
0.123153478	8.27744E-05	8.31707E+16
0.182242611	0.000418836	4.04082E+16
0.208276311	0.001076231	5.55107E+16
0.131933382	0.000318082	5.79348E+16
0.133684533	9.15727E-05	6.95306E+16
0.143362768	0.000287761	7.29708E+16
0.158418835	0.000176848	3.24603E+16
0.195274634	0.000353493	1.20158E+17
0.13963337	2.80346E-05	1.61316E+16
0.099209195	0.000213654	3.87277E+16
0.278251499	0.002540902	3.14054E+16
0.147538028	0.000499698	1.06559E+16
0.223004471	6.04995E-05	1.87986E+16
0.180452546	0.000432575	1.29444E+16
0.205141627	0.000470857	3.74373E+16
0.224309795	0.001122296	1.15327E+17
0.268332365	4.45003E-05	6.73983E+16
0.343503474	0.0002474	2.97199E+16
0.261612804	0.000111078	3.76673E+16
0.310638154	0.00207492	2.98654E+16
0.597819421	0.015380222	2.60858E+16
0.640715237	0.004771128	1173.578465
0.493833996	0.002549716	462.6978462
0.39810871	0.001407234	1345.892856
0.68326342	0.003710997	1.41441E+17
0.395765929	0.001239224	1078.823517
0.32547678	0.000712092	4.74438E+16
0.216862607	0.002115831	1.16629E+16
0.975823887	0.529668067	6.84146E+17
0.63137045	0.016053114	2.94228E+16
0.953142954	0.254401843	272.1824711
0.349007742	0.004742073	295.8359956
0.707771337	7.84967E-06	296.1363201
0.268529921	0.00029874	305.8976514
0.310137361	5.07118E-05	302.9322172
0.443490884	0.021827679	305.9754896
0.230218807	0.000497058	325.0210893
0.360862464	0.000886792	319.1632777
0.174175068	0.000751773	323.7547936
0.239116034	0.000607648	324.1368266
0.270130038	5.69562E-06	326.3806009
0.504580516	0.007306259	326.2927132
0.122927883	1.48386E-05	326.8112159
0.188943272	7.46013E-06	327.5590028
0.13680231	2.33454E-05	327.8192445
0.134641815	0.00013003	327.3266622
0.259976996	0.00055039	329.0547068
0.364674437	0.004539172	328.9158551
0.415594539	0.01470682	297.6870519

0.263630195	0.008927913	296.9997723
0.34853427	0.000168997	936.0474115
0.520828419	0.000315104	896.5753703
0.234390044	1.6794E-05	892.5681806
0.186527155	0.000360132	916.5486907
0.242635593	4.84846E-05	919.4917179
0.210696739	1.1568E-06	918.9555313
0.171558942	5.97438E-05	918.1168679
